# Genetic Dissection of Hybrid Performance and Heterosis for Yield-Related Traits in Maize

**DOI:** 10.3389/fpls.2021.774478

**Published:** 2021-11-30

**Authors:** Dongdong Li, Zhiqiang Zhou, Xiaohuan Lu, Yong Jiang, Guoliang Li, Junhui Li, Haoying Wang, Shaojiang Chen, Xinhai Li, Tobias Würschum, Jochen C. Reif, Shizhong Xu, Mingshun Li, Wenxin Liu

**Affiliations:** ^1^Key Laboratory of Crop Heterosis and Utilization, The Ministry of Education/Key Laboratory of Crop Genetic Improvement, Beijing Municipality/National Maize Improvement Center/College of Agronomy and Biotechnology, China Agricultural University, Beijing, China; ^2^Institute of Crop Science, Chinese Academy of Agricultural Sciences, Beijing, China; ^3^Leibniz Institute of Plant Genetics and Crop Plant Research (IPK), Stadt Seeland, Germany; ^4^Institute of Plant Breeding, Seed Science and Population Genetics, University of Hohenheim, Stuttgart, Germany; ^5^Department of Botany and Plant Sciences, University of California, Riverside, Riverside, CA, United States

**Keywords:** maize, hybrid performance, midparent heterosis, epistatic effect, pleiotropic loci, genomic selection

## Abstract

Heterosis contributes a big proportion to hybrid performance in maize, especially for grain yield. It is attractive to explore the underlying genetic architecture of hybrid performance and heterosis. Considering its complexity, different from former mapping method, we developed a series of linear mixed models incorporating multiple polygenic covariance structures to quantify the contribution of each genetic component (additive, dominance, additive-by-additive, additive-by-dominance, and dominance-by-dominance) to hybrid performance and midparent heterosis variation and to identify significant additive and non-additive (dominance and epistatic) quantitative trait loci (QTL). Here, we developed a North Carolina II population by crossing 339 recombinant inbred lines with two elite lines (Chang7-2 and Mo17), resulting in two populations of hybrids signed as Chang7-2 × recombinant inbred lines and Mo17 × recombinant inbred lines, respectively. The results of a path analysis showed that kernel number per row and hundred grain weight contributed the most to the variation of grain yield. The heritability of midparent heterosis for 10 investigated traits ranged from 0.27 to 0.81. For the 10 traits, 21 main (additive and dominance) QTL for hybrid performance and 17 dominance QTL for midparent heterosis were identified in the pooled hybrid populations with two overlapping QTL. Several of the identified QTL showed pleiotropic effects. Significant epistatic QTL were also identified and were shown to play an important role in ear height variation. Genomic selection was used to assess the influence of QTL on prediction accuracy and to explore the strategy of heterosis utilization in maize breeding. Results showed that treating significant single nucleotide polymorphisms as fixed effects in the linear mixed model could improve the prediction accuracy under prediction schemes 2 and 3. In conclusion, the different analyses all substantiated the different genetic architecture of hybrid performance and midparent heterosis in maize. Dominance contributes the highest proportion to heterosis, especially for grain yield, however, epistasis contributes the highest proportion to hybrid performance of grain yield.

## Introduction

Heterosis is the phenomenon that a hybrid outperforms its two parents (Birchler et al., 2006; Lippman and Zamir, 2007). Maize is the most successful example for the utilization of heterosis in crops to improve agricultural production, as single-cross varieties of maize have substantially contributed to the improvement of maize production in the past decades (Hochholdinger and Baldauf, 2018). There are three hypotheses to explain the genetic basis of heterosis: dominance (Bruce, 1910; Jones, 1917), overdominance (East, 1936) and epistasis (Powers, 1944). Many studies were performed to test these hypotheses, but the results often varied, depending on the populations and the traits studied, suggesting that heterosis is a complex genetic phenomenon. One commonly used design to study heterosis is the North Carolina Design III (NCIII) or Triple Testcross Design which allows to estimate the contribution of additive, dominance, and epistasis effects to heterosis (Melchinger et al., 2007b; Garcia et al., 2008). In a maize study, a total of 264 F_3_ genotypes were generated by intercrossing B73 and Mo17, and the F_3_ genotypes were then backcrossed to the two parents. The results showed that nearly all heterozygous individuals performed better than the homozygous individuals, supporting the overdominance (or pseudo-overdominance) hypothesis (Stuber et al., 1992). Conversely, the analysis of hybrid maize data from another NCIII design showed that dominance loci contributed the most to heterosis in maize, while the additive-by-additive effects contributed the most to the heterosis of rice (Garcia et al., 2008).

An alternative design is the North Carolina Design II (NCII) or factorial design, where a set of males is crossed with a set of females in a balanced or unbalanced way. In a partial NCII of maize, eight main effect (additive and dominance) QTL and 37 epistatic QTL pairs were identified (Bu et al., 2015). In addition to the NC mating designs, advanced maize populations were also developed and used for analysis of heterosis. For example, Wei et al. (2016) detected 36 heterotic loci from a series of single-segment substitution lines. Using near-isogenic lines for QTL detection, many additive QTL and additive-by-additive QTL pairs were detected (Melchinger et al., 2007a; Reif et al., 2009). An immortalized F_2_ population (IMF_2_) was also a promising mating design for dissecting the genetic basis of heterosis and epistasis QTL (Hua et al., 2003; Xu, 2013; Guo et al., 2014; Yi et al., 2019).

Linear mixed models (LMM) are a powerful tool for the genetic dissection of complex traits and are widely used in plant and animal breeding (Yu et al., 2006; Xu et al., 2014; Cui et al., 2020). In a hybrid population of rice, a LMM incorporating multiple polygenic covariance structures to control the genetic background was developed (Xu, 2013). In wheat, a quantitative genetics approach was proposed to dissect the genetic basis of grain-yield heterosis, allowing QTL mapping of dominance, epistasis and heterotic loci for midparent heterosis (MPH) (Jiang et al., 2017). In addition to QTL mapping, genomic selection (GS) has become a new tool for plant breeding and the genetic dissection of complex traits (Meuwissen et al., 2001) and has been applied to hybrid wheat (Zhao et al., 2013, 2015b; Jiang et al., 2017), hybrid rice (Cui et al., 2020) and hybrid maize (Albrecht et al., 2014; Technow et al., 2014).

The general combining ability (GCA) is a measure for the average performance of a line in different hybrid combinations, while the specific combining ability (SCA) describes the deviation of a hybrid from the performance expected based on the GCA of its two parental lines. The additive and additive-by-additive variances contribute to the variation of GCA, while the non-additive polygenic variances contribute to the variation of SCA (Reif et al., 2007). A two-step approach has been widely used to study the genetics underlying hybrid performance, where the first step consists of estimating the GCA, SCA and the MPH (Guo et al., 2014; Zhou et al., 2018; Yi et al., 2019) and the second step represents the QTL mapping step with the GCA, SCA and MPH treated as the traits of interest. In a previous genome-wide association study (GWAS) with an NCII population, different coding schemes for the genotypes were applied, namely the additive, dominance and recessive coding (Hyun et al., 2008; Liu et al., 2021). However, the additive model was usually not sufficient to explain hybrid performance and MPH. Thus, more elaborate models incorporating non-additive effects should be used to study heterosis.

In this study, we developed a NCII population of maize by crossing a set of 339 recombinant inbred lines (RILs) with two elite inbred lines, resulting in two populations of hybrids. A total of 10 traits were recorded in four to five environments and high-density genotypic data were obtained by genotyping-by-sequencing of the RILs and resequencing of the parents. The aims of this study were to (1) evaluate the heritability of MPH and the relative contribution of various traits to grain yield, (2) perform QTL mapping for main (additive and dominance) and non-additive effect loci for hybrid performance and MPH, (3) identify QTL hotspots for yield-related traits, (4) explore the mechanisms of heterosis and hybrid performance, and (5) assess the accuracy of genomic prediction in various breeding schemes.

## Materials and Methods

### Plant Materials

A RIL population consisting of 365 F_11_ lines was developed by crossing inbred lines Qi319 as the male parent and Ye478 as the female parent originating from two different heterotic groups of maize (Zhou et al., 2016). Two hybrid populations were developed by crossing the RILs to two female testers, Chang7-2 and Mo17, and the two populations Chang7-2 × RIL and Mo17 × RIL were named TC and TM, respectively (Zhou et al., 2018). Different numbers of offspring were obtained from the two hybrid populations. A total of 339 common lines from the RIL, TC, and TM populations were retained for further analysis.

### Experimental Design and Phenotypic Evaluation

The RIL, TC, and TM populations, their parents and the hybrids (Chang7-2 × Qi319, Mo17 × Qi319, Chang7-2 × Ye478, and Mo17 × Ye478) were field-evaluated in two different locations, Xinxiang (35.19°N and 113.53°E) and Shijiazhuang (37.27°N and 113.30°E), in two consecutive years, 2015 and 2016, resulting in 2 × 2 = 4 different environments. Traits recorded include plant height (PH, cm), ear height (EH, cm), row number per ear (RNPE, count), kernel number per row (KNPR, count), kernel thickness (KT, mm), kernel width (KW, mm), kernel length (KL, mm), volume weight (VW, g/L), hundred grain weight (HGW, g) and grain yield per plant (GY, g). Furthermore, in 2017, the VW trait was evaluated in the RIL population, traits HGW and GY were evaluated in all the three populations (RIL, TC, and TM) in one of the two locations, Xinxiang. Detailed descriptions of the traits evaluated can be found in a previous study (Lu et al., 2020).

We used a randomized incomplete block design with two replicates in each environment. To avoid competition, the RIL and the hybrid populations were planted separately. Each genotype was planted in two rows with a row interval of 0.6 m, a row length of 4 m and a plant interval of 0.25 m.

### Phenotypic Data Analysis

The combination between year and location was considered as an environment (a total of 4 or 5 environments). The studentized residual razor method (Bernal-Vasquez et al., 2016) was used to remove outliers with a threshold of 2.8. The best linear unbiased estimations (BLUE) of the fixed effects and the variance components of the random effects were estimated using the following model:

$$y_{i\,j\,k}=\mu +G_{i}+E_{j}+G*E_{i\,j}+R_{k}\,\left(E_{j}\right)+\varepsilon_{i\,j\,k},$$

where *y*_*ijk*_ was the phenotypic value of the *k*th replicate of genotype *i* from the *j*th environment, μ was the overall mean, *G*_*i*_ (*i* = 1, 2,…, 339) was the effect of the *i*th genotype, *E*_*j*_ (*j* = 1, 2,…, 5) was the effect of the *j*th environment, G*E_*ij*_ was the genotype-by-environment interaction effect, *R*_*k*_(*E*_*j*_) (*k* = 1, 2) was the effect of the *k*th replicate nested in the *j*th environment, ε_*ijk*_ was the residual. For estimation of variance components, all random effects were assumed to be normally distributed with mean 0 and variances denoted by $\sigma_{G}^{2}$, $\sigma_{G\times E}^{2}$ and $\sigma_{\varepsilon }^{2}$ for *G*_*i*_, G*E_*ij*_ and ε_*ijk*_, respectively. The broad-sense heritability of a trait was defined as (Falconer and Mackay, 1996),

$$H^{2}=\frac{\sigma_{G}^{2}}{\sigma_{G}^{2}+\frac{\sigma_{G\,\text{×}\,E}^{2}}{N_{E}}+\frac{\sigma_{\varepsilon }^{2}}{N_{E}\times N_{R}}},$$

where *N*_*E*_ = 4 or 5 was the number of environments and *N*_*R*_ = 2 was the number of replicates within each environment.

Genetic analysis of MPH was conducted in two steps (Jiang et al., 2017). The first step was represented by BLUE of the trait value for each parent and each hybrid. The BLUE of the trait value obtained from the two replicates in one environment was calculated with the following formula:

$$y_{i\,k}=\mu +G_{i}+R_{k}+\varepsilon_{i\,k},$$

where *y*_*ik*_ was the trait value for the *k*th replicate of genotype *i*, μ was the mean of the trait under the current environment, *G*_*i*_ was the genetic value of the *i*th genotype and *R*_*k*_ was the effect of the *k*th replicate assumed to follow a $N\,\left(0,\sigma_{R}^{2}\right)$ distribution, ε_*ik*_ was assumed to follow a $N\,\left(0,\sigma_{i\,k}^{2}\right)$ distribution.

The MPH was defined as (Melchinger et al., 2007b):

$$M\,P\,H=H-\left(P_{1}+P_{2}\right)/2.$$

Where *H* was the BLUE value of hybrids, *P*_1_ was the BLUE value of Chang7-2 or Mo17 (corresponding to female parent of hybrid), *P*_2_ was the BLUE value of RIL (corresponding to the male parent of hybrid).

The second step in the MPH analysis required the following mixed model:

$$M\,P\,H_{i\,j}=\mu +G_{i}+E_{j}+\varepsilon_{i\,j},$$

where *MPH*_*ij*_ was the MPH value calculated in the first step for hybrid (genotype) *i* in environment *j*, *G*_*i*_ (*i* = 1, 2, …, 339) was the genetic effect of MPH for the *i*th hybrid, *E*_*j*_ was effect of the *j*th environment and ε_*ij*_ was the residual. Noted that *G*_*i*_ was treated as a fixed effect in the BLUE calculation or a random effect following a $N\,\left(0,\sigma_{G}^{2}\right)$ distribution in variance estimation, *E*_*j*_ was treated as a random effect following a $N\,\left(0,\sigma_{E}^{2}\right)$ distribution and ε_*ij*_ was assumed to be $N\,\left(0,\sigma_{\varepsilon }^{2}\right)$ distributed. The variance components of the above linear mixed model were implemented using the ASReml 3.0 package in R (Gilmour et al., 2009).

In addition, the hybrid performance was decomposed into GCA, SCA and interaction with the environment using a two-step method. In the first step, the BLUEs in RIL, TC and TM populations were calculated within each environment following the same formula above. In the second step, the following formula was applied to the hybrid performance (Zhao et al., 2015a):

$$y=\mu +E+G\,C\,A_{R\,I\,L}+G\,C\,A_{T\,e\,s\,t\,e\,r}+S\,C\,A+G\,C\,A_{R\,I\,L}*E+G\,C\,A_{T\,e\,s\,t\,e\,r}*E+S\,C\,A*E+\varepsilon .$$

Where *y* was the hybrid performance, μ was the mean, *E* was the environment effect, *GCA*_*RIL*_ was the GCA of RILs, *GCA*_*Tester*_ was the GCA of testers, the rest was the interaction between GCA, SCA, and environment, ε was the error. All effects were treated as random following normal distributions. The variances were estimated in ASReml 3.0 package in R (Gilmour et al., 2009).

Path analysis can be used to determine the relative contribution of independent variables to a response variable. Path analysis was implemented in the R package sem by taking GY as the response variable and the other traits as independent variables. Path coefficient *p*_*i*_ of variable *X*_*i*_ was obtained by $p_{i}=b_{i}\,\sqrt{S\,S_{X_{i}}\,/\,S\,S_{Y}}$, where *b*_*i*_ was the partial correlation, *SS*_*X*_*i*__ and *SS*_*Y*_ were sum of square for *X*_*i*_ and the response variable *Y*, respectively. Path diagrams were drawn with the semPlot package in R, where values above 0.14 (*p* = 0.01, *n* = 339) were displayed.

### Genomic Data Analyses

The genotyping procedures for the RILs, the two parents of the RILs, and the two testers were described in a previous study (Zhou et al., 2016). In brief, for the four parents, the paired-end sequencing libraries were created with a fragment length of ∼500 bp and were sequenced on an Illumina HiSeq 2000 sequencer. The resequencing depth was ∼30×. For the RILs, a genotyping-by-sequencing (GBS) strategy was applied. A total of 137,699,000 reads were generated. On average, there were 357,376 reads per individual, which was approximately a 0.07-fold coverage of the maize genome. The cleaned reads were obtained after quality control.

The filtered high-quality reads of the four parents and the RILs were mapped to the reference genome (B73_RefGen_v4) with BWA (Li and Durbin, 2009). SAMtools (Li, 2011) were used to call SNPs with quantity over 20 and a total of 41,791,163 SNPs were finally produced. Details regarding the parameters for the SNP calling process can be found in a previous report (Zhou et al., 2016). After filtering of all SNPs for minor allele frequency < 0.05, missing rate > 0.1 and unknown physical positions, a total of 36,095 SNPs remained in the data set for analysis. Missing genotypes of SNPs were imputed using the BEAGLE software package (version v5) with the default parameters (Browning and Browning, 2016).

The low-coverage high-throughput sequence technologies like GBS generate sequences that are often error-prone, which might lead to errors for detection of genetic variants (Ma et al., 2019). Therefore, the hmm.vitFUN.rils function in the R package MPR.genotyping was used to correct the genotyping errors using a Hidden Markov model with errorRate = 0.05 (Xie et al., 2010). The SNPs with high error probabilities were either corrected or set to missing values.

The bin function in the ICIMapping package was used to bin redundant markers with missing rate > 0.2 and a distortion *p*-value < 0.001, while missing values and anchor information were considered at the same time (Meng et al., 2015). After the above imputation and correction, there were still a little proportion of missing values left, then the argmax method in qtl/R was used to perform the final imputation additive-by-dominance and (Broman et al., 2003). Finally, a total of 4,141 bins were discovered across the entire maize genome. The genetic map was constructed using the map function in the IciMapping package with the default parameter values (Meng et al., 2015).

### Mapping Quantitative Trait Loci in Recombinant Inbred Line, TC, TM, and Pooled TC-TM Populations

To determine the contribution of each genetic component to hybrid performance and MPH variation and identify significant non-additive QTL, firstly, we combined the TC and TM populations to form a pooled population called TC-TM. For 341 lines (337RILs, 2 parents; 2 testers) lines, if the genotypes were the same as Ye478, it was coded as “1”; if the genotypes were the same as Qi319, it was coded as “−1”, then the genotypes of the hybrids were inferred from their parents (the RILs and the testers). The additive and dominance coding matrices, *Z* and *W*, for individual *j* at marker *k* were coded as *Z*_*jk*_ = {1 0 −1} for the additive effect and *W*_*jk*_ = {0 1 0} for the dominance effect.

The linear mixed model for variance component analysis was (Xu, 2013; Jiang et al., 2017):

(1) $$y=X\,\beta +\xi_{a}+\xi_{d}+\xi_{a\,a}+\xi_{a\,d}+\xi_{d\,d}+\varepsilon ,$$

where *y* was an *n*×1 vector of phenotypic values of the hybrids and *X*β captured the fixed effects of the model that were not relevant to genetic effects. The design matrix for the fixed effects was *X* = [*X*_0_,*X*_1_], where *X*_0_ was an *n*×1 vector of unity (a vector with all elements being 1) and *X*_1_ was an *n*×1 vector indicating one of the two populations, *X*_*j*1_ = 0 for TC and *X*_*j*1_ = 1 for TM. The last term of model (1) was a vector of residuals. The remaining terms in model (1) were various polygenic effects (each polygenic effect was an *n*×1 vector) and were defined below. $\xi_{a}=\sum_{k=1}^{m}Z_{k}\,a_{k}$ was the polygenic additive effect; $\xi_{d}=\sum_{k=1}^{m}W_{k}\,d_{k}$ was the polygenic dominance effect; $\xi_{a\,a}=\sum_{k=1}^{m-1}\sum_{k^{\prime }=k+1}^{m}\left(Z_{k}\,\#\,Z_{k^{\prime }}\right)\,{\left(a\,a\right)}_{k\,k^{\prime }}$ was the polygenic additive-by-additive effect; $\xi_{a\,d}=\sum_{k,k^{\prime }=1,k^{\prime }\neq k}^{m}\left(Z_{k}\,\#\,W_{k^{\prime }}\right)\,{\left(a\,d\right)}_{k\,k^{\prime }}$ was the polygenic additive-by-dominance effect; $\xi_{d\,d}=\sum_{k=1}^{m-1}\sum_{k^{\prime }=k+1}^{m}\left(W_{k}\,\#\,W_{k^{\prime }}\right)\,{\left(d\,d\right)}_{k\,k^{\prime }}$ was the polygenic dominance-by-dominance effect. The operator _#_ represented element-wise product of matrices. In the formulas above, *a*_*k*_ and *d*_*k*_ were the additive and dominance effect for marker *k*, (*aa*)*kk*′, (*ad*)*kk*′, and (*dd*)*kk*′ were the additive-by-additive, additive-by-dominance and dominance-by-dominance effect between markers *k* and *k*’, respectively. The distributions for the polygenic and residual effects were $\xi_{a}∼N\,\left(0,K_{a}\,\sigma_{a}^{2}\right)$, $\xi_{d}∼N\,\left(0,K_{d}\,\sigma_{d}^{2}\right)$, $\xi_{a\,a}∼N\,\left(0,K_{a\,a}\,\sigma_{a\,a}^{2}\right)$, $\xi_{a\,d}∼N\,\left(0,K_{a\,d}\,\sigma_{a\,d}^{2}\right)$, $\xi_{d\,d}∼N\,\left(0,K_{d\,d}\,\sigma_{d\,d}^{2}\right)$, and ε∼*N*(0,*I*σ^2^), where *K*_*a*_, *K*_*d*_, *K*_*aa*_, *K*_*ad*_, and *K*_*dd*_ were the corresponding kinship matrices calculated using the method given by Xu (2013). The six variance components (five genetic variance components and the residual variance) were estimated using the BGLR package in R (Pérez and De Los Campos, 2014) with the number of iterations set at 15,000 and the number of burn-in set at 5,000.

The variance-covariance matrix of *y* was

$$v\,a\,r\,\left(y\right)=K_{a}\,\sigma_{a}^{2}+K_{d}\,\sigma_{d}^{2}+K_{a\,a}\,\sigma_{a\,a}^{2}+K_{a\,d}\,\sigma_{a\,d}^{2}+K_{d\,d}\,\sigma_{d\,d}^{2}+I\,\sigma^{2}.$$

Let $\lambda_{x}=\sigma_{x}^{2}/\sigma^{2}$, where $\sigma_{x}^{2}$ was one of the five genetic variance components and σ^2^ was the residual variance. The above variance could be rewritten as

$$v\,a\,r\,\left(y\right)=\left(K_{a}\,\lambda_{a}+K_{d}\,\lambda_{d}+K_{a\,a}\,\lambda_{a\,a}+K_{a\,d}\,\lambda_{a\,d}+K_{d\,d}\,\lambda_{d\,d}+I\right)\,\sigma^{2}.$$

Define

$$K=K_{a}\,\lambda_{a}+K_{d}\,\lambda_{d}+K_{a\,a}\,\lambda_{a\,a}+K_{a\,d}\,\lambda_{a\,d}+K_{d\,d}\,\lambda_{d\,d},$$

so that

$$v\,a\,r\,\left(y\right)=\left(K+I\right)\,\sigma^{2}.$$

Let

$$\xi =\xi_{a}+\xi_{d}+\xi_{a\,a}+\xi_{a\,d}+\xi_{d\,d}.$$

Model (1) could be rewritten as

(2) y=X⁢β+ξ+ε,

which was the null model for the GWAS of main effect and epistatic effect detection. On this null model, we added a specific marker or marker pair to the model to test the putative effect.

To test the additive effect of marker *k*, we added *Z*_*k*_*a*_*k*_ to the null mode so that the linear mixed model became:

(3) $$y=X\,\beta +Z_{k}\,a_{k}+\xi +\varepsilon .$$

Let *e* = *ξ* + ε, so that the model was rewritten as:

(4) $$y=X\,\beta +Z_{k}\,a_{k}+e.$$

The expectation of model (4) was *E*(*y*) = *X*β + *Z*_*k*_*a*_*k*_ and the variance was:

$$v\,a\,r\,\left(y\right)=v\,a\,r\,\left(e\right)=v\,a\,r\,\left(\xi +\varepsilon \right)=\left(K+I\right)\,\sigma^{2}.$$

Let us perform eigenvalue decomposition for matrix *K*, *K* = *UD*^*UT*^, where *U* was the eigenvector matrix and *D* was a diagonal matrix holding the eigenvalues. So,

$$v\,a\,r\,\left(e\right)=U\,\left(D+I\right)\,U^{T}\,\sigma^{2}.$$

Let $Q^{T}=\sqrt{{\left(D+I\right)}^{-1}}\,U^{T}$ and pre-multiply equation (4) by *Q*^*T*^ leading to

(5) $$Q^{T}\,y=Q^{T}\,\left(X\,\beta +Z_{k}\,a_{k}+e\right)=Q^{T}\,X\,\beta +Q^{T}\,Z_{k}\,a_{k}+Q^{T}\,e.$$

Let *y*^∗^ = *Q^T^y*, *X*^∗^ = *Q^T^X*, $Z_{k}^{*}=Q^{T}\,Z_{k}$ and *e*^∗^ = *Q*^*T*^*e*. The above linear mixed model was

(6) $$y^{*}=X^{*}\,\beta +Z_{k}^{*}\,a_{k}+e^{*}.$$

The variance of the transformed residuals was

$$\begin{array}{c} v\,a\,r\,\left(e^{*}\right)=v\,a\,r\,\left(Q^{T}\,e\right)\\ =Q^{T}\,U\,\left(D+I\right)\,U^{T}\,Q\,\sigma^{2}\\ =\sqrt{{\left(D+I\right)}^{-1}}\,U^{T}\,U\,\left(D+I\right)\,U^{T}\,U\,\sqrt{{\left(D+I\right)}^{-1}}\,\sigma^{2}\\ =\sqrt{{\left(D+I\right)}^{-1}}\,\left(D+I\right)\,\sqrt{{\left(D+I\right)}^{-1}}\,\sigma^{2}\\ =\sqrt{{\left(D+I\right)}^{-1}}\,\sqrt{\left(D+I\right)}\,\sqrt{\left(D+I\right)}\,\sqrt{{\left(D+I\right)}^{-1}}\,\sigma^{2}\\ =I\,\sigma^{2}. \end{array}$$

The expectation and variance of *y*^∗^ were $E\,\left(y^{*}\right)=X^{*}\,\beta +Z_{k}^{*}\,a_{k}$ and *var*(*y*^∗^) = *I*σ^2^. Therefore, model (6) became a simple linear model with a homogeneous residual variance. The conventional least squares method could be used to estimate the parameters and test for the marker effect. Since the model of the transformed phenotypic values was very simple, the “lm” function in R was applied to estimate the marker effect and test the significance of the marker.

Considering the dominance and epistatic effects, we adopted a more general likelihood ratio test (LRT) for a particular effect. The likelihood ratio test for the additive effect of marker *k* was

$$L\,R\,T=-2\,\left[L_{0}\,\left(\hat{\beta }\right)-L_{1}\,\left(\hat{\beta },{\hat{a}}_{k}\right)\right],$$

where $L_{0}\,\left(\hat{\beta }\right)$ was the likelihood value evaluated from the null model given in equation (7) below,

(7) $$y^{*}=X^{*}\,\beta +e^{*},$$

and $L_{1}\,\left(\hat{\beta },{\hat{\alpha }}_{k}\right)$ was the likelihood value evaluated from the full model given in equation (6). The LRT statistic was eventually converted into the log of odds (LOD) score using *LOD* = *LRT*/4.61. If the intervals of different QTL were overlapped or the genetic distance of peak SNP of two QTL was within 0.65 cM (the average density in the whole genome), we called such QTL as a pleiotropic QTL (a QTL affecting more than one trait).

Dominance effect of marker *k* was detected using the same model as the additive effect except that *Z*_*k*_ was replaced by *W*_*k*_. In the following, we called the significant additive and dominance QTL as the main effect QTL.

The additive-by-additive effect was detected by the following likelihood ratio test,

$$L\,R\,T=-2\,\left[L_{0}\,\left(\hat{\beta },{\hat{a}}_{k},{\hat{a}}_{k^{\prime }}\right)-L_{1}\,\left(\hat{\beta },{\hat{a}}_{k},{\hat{a}}_{k^{\prime }},{\left(a\,a\right)}_{k\,k^{\prime }}\right)\right],$$

where the null model was

(8) $$y^{*}=X^{*}\,\beta +Z_{k}^{*}\,a_{k}+Z_{k^{\prime }}^{*}\,a_{k^{\prime }}+e^{*},$$

and the full model was

(9) $$y^{*}=X^{*}\,\beta +Z_{k}^{*}\,a_{k}+Z_{k^{\prime }}^{*}\,a_{k^{\prime }}+\left(Z_{k}^{*}\,\#\,Z_{k^{\prime }}^{*}\right)\,{\left(a\,a\right)}_{k\,k^{\prime }}+e^{*},$$

Similarly, the additive-by-dominance effect was detected using

$$L\,R\,T=-2\,\left[L_{0}\,\left(\hat{\beta },{\hat{a}}_{k},{\hat{d}}_{k^{\prime }}\right)-L_{1}\,\left(\hat{\beta },{\hat{a}}_{k},{\hat{d}}_{k^{\prime }},{\left(a\,d\right)}_{k\,k^{\prime }}\right)\right].$$

The null model and the full model were

(10) $$y^{*}=X^{*}\,\beta +Z_{k}^{*}\,a_{k}+W_{k^{\prime }}^{*}\,d_{k^{\prime }}+e^{*},$$

and

(11) $$y^{*}=X^{*}\,\beta +Z_{k}^{*}\,a_{k}+W_{k^{\prime }}^{*}\,d_{k^{\prime }}+\left(Z_{k}^{*}\,\#\,W_{k^{\prime }}^{*}\right)\,{\left(a\,d\right)}_{k\,k^{\prime }}+e^{*},$$

respectively. Similarly, the dominance-by-additive effect was detected using

$$L\,R\,T=-2\,\left[L_{0}\,\left(\hat{\beta },{\hat{a}}_{k},{\hat{d}}_{k^{\prime }}\right)-L_{1}\,\left(\hat{\beta },{\hat{a}}_{k},{\hat{d}}_{k^{\prime }},{\left(d\,a\right)}_{k\,k^{\prime }}\right)\right].$$

The null model and the full model were

(12) $$y^{*}=X^{*}\,\beta +Z_{k}^{*}\,a_{k}+W_{k^{\prime }}^{*}\,d_{k^{\prime }}+e^{*},$$

and

(13) $$y^{*}=X^{*}\,\beta +Z_{k}^{*}\,a_{k}+W_{k^{\prime }}^{*}\,d_{k^{\prime }}+\left(W_{k}^{*}\,\#\,Z_{k^{\prime }}^{*}\right)\,{\left(d\,a\right)}_{k\,k^{\prime }}+e^{*},$$

respectively. Finally, the dominance-by-dominance effect was tested using

$$L\,R\,T=-2\,\left[L_{0}\,\left(\hat{\beta },{\hat{d}}_{k},{\hat{d}}_{k^{\prime }}\right)-L_{1}\,\left(\hat{\beta },{\hat{d}}_{k},{\hat{d}}_{k^{\prime }},{\left(d\,d\right)}_{k\,k^{\prime }}\right)\right].$$

The corresponding null model and full model were

(14) $$y^{*}=X^{*}\,\beta +W_{k}^{*}\,d_{k}+W_{k^{\prime }}^{*}\,d_{k^{\prime }}+e^{*},$$

and

(15) $$y^{*}=X^{*}\,\beta +W_{k}^{*}\,d_{k}+W_{k^{\prime }}^{*}\,d_{k^{\prime }}+\left(W_{k}^{*}\,\#\,W_{k^{\prime }}^{*}\right)\,{\left(d\,d\right)}_{k\,k^{\prime }}+e^{*},$$

respectively. LOD scores were converted the same way as we did for the additive effect.

An empirical threshold of 2.5 for the LOD score was used to determine significance of an additive or a dominance effect. A LOD threshold of 5.0 was used to determine the significance of an epistatic effect (Churchill and Doerge, 1994; Xu, 2013). A confidence interval in the genome was determined for each detected QTL with the following steps: (1) all significant SNPs passing the threshold were selected; (2) the most significant SNPs were kept within a 10 cM interval; (3) the QTL interval was formed using a 1.5-LOD drop-off method (Broman, 2001). The names of QTL referred to McCouch’s method (McCouch et al., 1997), and a dash (–) was added to designate different datasets.

The estimated additive and dominance effects for each QTL were extracted from the estimated regression coefficients (*a*_*k*_ and *d*_*k*_) from the models presented above. The proportion of the phenotypic variance explained (PVE) contributed by each QTL was calculated using (Utz et al., 2000; Garin et al., 2017),

$$P\,V\,E=1-\frac{R\,S\,S_{F\,u\,l\,l}}{R\,S\,S_{N\,u\,l\,l}},$$

where *RSS*_*Full*_ was the residual sum of squares of the full model and *RSS*_*Null*_ was the residual sum of squares of the null model.

We also performed QTL mapping in the RIL, TC and TM population separately. The model was the same as described above except that only the additive and additive-by-additive polygenic effects were used to control genetic background. QTL mapping for MPH was conducted using a similar linear mixed model to the original traits. Details of the MPH analysis can be found in a previous study (Jiang et al., 2017).

### Genomic Selection

The genetic effects of single-cross hybrids can be dissected into additive, dominance and epistatic polygenic effects as mentioned before. Here, we only considered the first two components in the genomic prediction model. The linear mixed model was (Su et al., 2012; Xu et al., 2014).

$$y=X\,\beta +\xi_{a}+\xi_{d}+\varepsilon ,$$

where *y* was the phenotype vector, *X*β represented the fixed effect, *ξ*_*a*_ was the additive polygenic effect with an assumed distribution of $\xi_{a}∼N\,\left(0,K_{a}\,\sigma_{a}^{2}\right)$, *ξ*_*d*_ was the dominance polygenic effect with a distribution of $\xi_{d}∼N\,\left(0,K_{d}\,\sigma_{d}^{2}\right)$, *K*_*a*_ was the additive kinship matrix and *K*_*d*_ was the dominance kinship matrix.

Three genomic prediction schemes were proposed to mimic the scenarios in practical genomic hybrid breeding. Scheme (1), abbreviated as CV1: to predict the trait values for the TM population from the phenotypes and genotypes of the TC population or vice versa. Scheme (2), abbreviated as CV2: to select the hybrids sharing the same RILs in TC and TM population as the training set to predict the rest of the population. Scheme (3), abbreviated as CV3: to select the hybrids having the different RILs in TC and TM population as the training set to predict the rest of the population. Scheme (1) and (2) belong to the so-called T1 case, and scheme (3) is in the category of T2 (Technow et al., 2014; Zhao et al., 2015a). The three scenarios are illustrated in Supplementary Figure 1.

The across population prediction in scheme (1) was conducted using a model that contained only the additive polygenic effect. For schemes (2) and (3), the prediction models contained both the additive and the dominant polygenic effects. The predictions were implemented with the BGLR software package in R (Pérez and De Los Campos, 2014). The prediction accuracy was assessed with a two-fold cross-validation scheme. In each run, 1/2 of the lines were removed from the training set and then the correlation between the predicted values and the observed values of the removed lines was calculated. The two-fold cross-validation was repeated 200 times. In addition, significant SNPs were treated as fixed effects in the prediction model, which has been termed wGS (Bernardo, 2014; Würschum et al., 2018). For example, when the TC population was used to predict the TM population, the QTL detected in the TC population were treated as fixed effects in the linear mixed model used to predict the TM population. For schemes (2) and (3), QTL detected from the pooled population of TC and TM were treated as fixed effects included in the models to predict the rest of the population. For comparison, the additive model with kinship matrix inferred from RILs population was used to yield the prediction accuracy of 10 traits using a two-fold cross-validation scheme in TC and TM population, respectively. This process was repeated 200 times. Data visualization was done with the ggplot2 and ggpubr packages in R (Wickham, 2016).

## Results

### Phenotypic Variation and Heritability in the Recombinant Inbred Line, TC, and TM Populations

**FIGURE 1:**
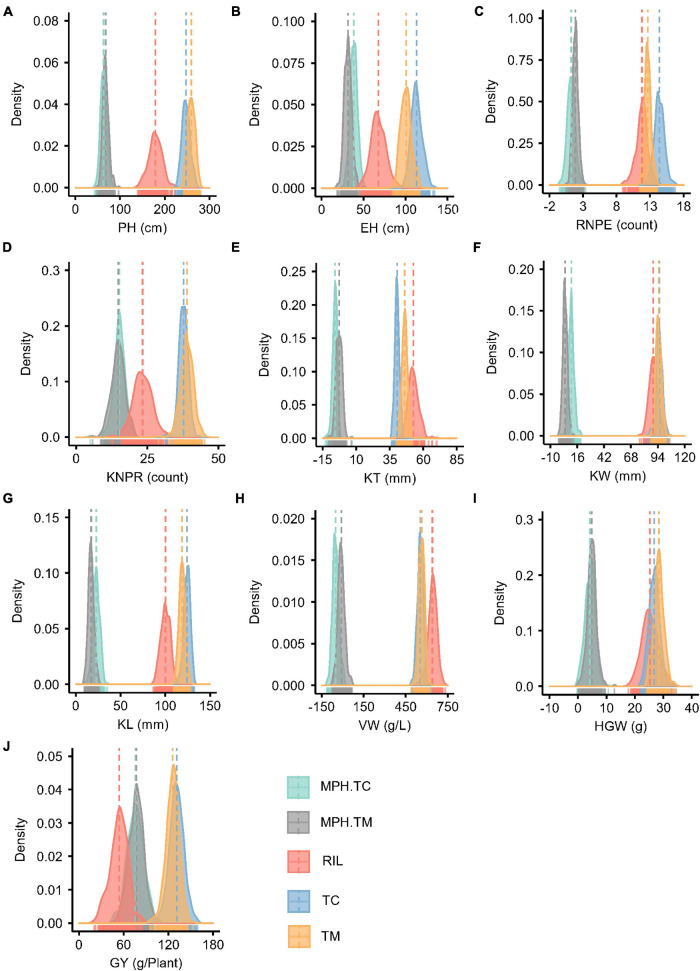
Phenotype and midparent heterosis (MPH) distributions for 10 traits in the recombinant inbred line population developed by Ye478 × Qi319 (RIL), Chang7-2 × RIL (TC), and Mo17 × RIL (TM) populations. **(A)** PH, plant height; **(B)** EH, ear height; **(C)** RNPE, row number per ear; **(D)** KNPR, kernel number per row; **(E)** KT, kernel thickness; **(F)** KW, kernel width; **(G)** KL, kernel length; **(H)** VW, volume weight; **(I)** HGW, hundred grain weight; **(J)** GY, grain yield per plant; MPH.TC, MPH in TC population; MPH.TM, MPH in TM population.

The RIL population showed a larger variation for the 10 investigated traits than the TC and TM populations (Table 1 and Figure 1). The genetic variance components were significant (*p* < 0.01) for all traits in the three populations. Except for the trait VW in the TM population, the variance of the genotype-by-environment interaction was also significant (*p* < 0.01) for all traits. The estimated broad-sense heritability ranged from 0.68 for VW to 0.95 for PH in the RIL population, from 0.57 for VW to 0.91 for PH and KT in the TC population, and from 0.60 for GY to 0.89 for PH in the TM population. In general, PH had the highest heritability and VW or GY had the lowest heritability. The obtained moderate to high heritability implied that the experimental designs and phenotyping procedures were appropriate and accurate.

**TABLE 1:** Summary statistics for 10 traits in the recombinant inbred line population developed by Ye478 × Qi319 (RIL), Chang7-2 × RIL (TC), and Mo17 × RIL (TM) populations.

Population	Traits	Min	Max	Mean	*SD*	CV (%)	$\sigma_{G}^{2}$	$\sigma_{G\,\text{*}\,E}^{2}$	$\sigma_{\varepsilon }^{2}$	*N*_*E*_	*H* ^2^
RIL	PH	138.88	223.17	179.28	15.29	8.53	217.97**	26.64**	45.08	4	0.95
	EH	45.48	94.49	67.87	8.91	13.13	73.83**	9.39**	17.18	4	0.94
	RNPE	8.91	14.41	11.79	0.90	7.63	0.71**	0.13**	0.34	4	0.90
	KNPR	12.57	32.21	23.31	3.25	13.95	8.69**	3.72**	4.03	4	0.86
	KT	40.49	69.81	52.59	3.98	7.57	13.40**	2.84**	9.54	4	0.88
	KW	76.29	104.84	89.60	4.26	4.75	15.28**	3.07**	13.34	4	0.86
	KL	86.65	115.59	100.17	5.14	5.13	21.96**	5.76**	17.26	4	0.86
	VW	509.00	732.26	640.84	32.39	5.05	606.28**	399.41**	2026.25	5	0.68
	HGW	17.68	33.72	25.27	2.90	11.46	7.30**	2.41**	4.70	5	0.88
	GY	20.47	84.90	53.83	11.76	21.86	113.05**	59.47**	65.54	5	0.86
TC	PH	204.20	271.68	247.23	9.34	3.78	77.97**	8.67**	42.30	4	0.91
	EH	98.15	134.09	113.17	6.49	5.73	35.49**	5.53**	28.35	4	0.88
	RNPE	12.47	16.74	14.36	0.73	5.05	0.44**	0.07**	0.46	4	0.86
	KNPR	31.36	43.04	37.74	1.59	4.22	2.07**	1.00**	3.42	4	0.75
	KT	36.29	46.66	40.44	1.72	4.25	3.18**	0.15**	2.29	4	0.91
	KW	84.78	104.00	95.24	3.02	3.17	7.15**	2.16**	8.99	4	0.81
	KL	111.28	132.37	124.22	3.63	2.92	9.22**	4.60**	17.58	4	0.73
	VW	489.34	633.25	555.35	21.81	3.93	252.96**	143.69**	1238.20	4	0.57
	HGW	22.09	34.48	26.77	1.87	6.98	2.47**	1.33**	5.11	5	0.76
	GY	99.01	158.78	131.15	9.68	7.38	53.37**	35.86**	235.67	5	0.63
TM	PH	212.26	280.26	259.11	8.61	3.32	65.16**	15.32**	31.10	4	0.89
	EH	84.30	117.25	100.55	6.27	6.24	33.39**	8.11**	20.62	4	0.88
	RNPE	11.44	14.06	12.66	0.48	3.77	0.18**	0.03**	0.23	4	0.83
	KNPR	29.00	45.17	38.95	2.22	5.69	3.44**	2.86**	4.20	4	0.74
	KT	39.84	57.03	46.04	2.17	4.72	3.79**	0.69**	4.65	4	0.83
	KW	87.14	105.99	94.79	2.76	2.91	6.02**	0.98**	8.00	4	0.83
	KL	109.41	128.48	118.74	3.43	2.89	8.62**	1.98**	15.39	4	0.78
	VW	491.34	627.42	563.82	20.76	3.68	237.35**	68.33	1102.22	4	0.61
	HGW	24.16	34.25	28.39	1.75	6.16	2.17**	1.31**	4.68	5	0.75
	GY	94.99	152.12	125.65	8.74	6.95	40.76**	45.49**	180.99	5	0.60

SD, standard deviation; CV, coefficient of variation; $\sigma_{G}^{2}$, genotypic variance; $\sigma_{G\,\text{*}\,E}^{2}$, genotype-by-environment interaction variance; $\sigma_{\varepsilon }^{2}$, error variance; *N*_*E*_, the number of environments; *H*^2^, broad-sense heritability; **, significance at 0.01 level; PH, plant height; EH, ear height; RNPE, row number per ear; KNPR, kernel number per row; KT, kernel thickness; KW, kernel width; KL, kernel length; VW, volume weight; HGW, hundred grain weight; GY, grain yield per plant.

The MPH for traits KT and VW was negative on average in both the TC and TM populations, which means that the hybrids often had phenotypic values lower than the mean of the two parents (Figure 1). In both the TC and TM populations, GY had the highest heterosis, followed by PH and EH (Supplementary Table 1), and the genetic variances of MPH were statistically significant for all traits. The heritability of MPH in the TC population ranged from 0.36 for VW to 0.81 for PH, while the heritability of MPH ranged from 0.27 for VW to 0.78 for PH in the TM population. The moderate to high heritability of MPH lay the foundation to dissect the genetic architecture of heterosis.

For PH, EH, RNPE, KT, and KL the GCA variance of testers ($\sigma_{G\,C\,A_{T\,e\,s\,t\,e\,r}}^{2}$) had larger values than the GCA variance of RILs ($\sigma_{G\,C\,A_{R\,I\,L}}^{2}$), which indicated that the testers played an important role in hybrid performance. The SCA/GCA ratios indicating a relative contribution of additive and non-additive (dominance and epistasis) effects to phenotypic variation ranged from 0.04 for KT and 0.77 for GY (Supplementary Table 2). And for GY, the variance of SCA ($\sigma_{S\,C\,A}^{2}$) was higher than both $\sigma_{G\,C\,A_{T\,e\,s\,t\,e\,r}}^{2}$ and $\sigma_{G\,C\,A_{R\,I\,L}}^{2}$, which was consistent with the large MPH variation in phenotype (Figure 1J).

### Trait Correlation and Path Analysis in the Three Populations

**FIGURE 2:**
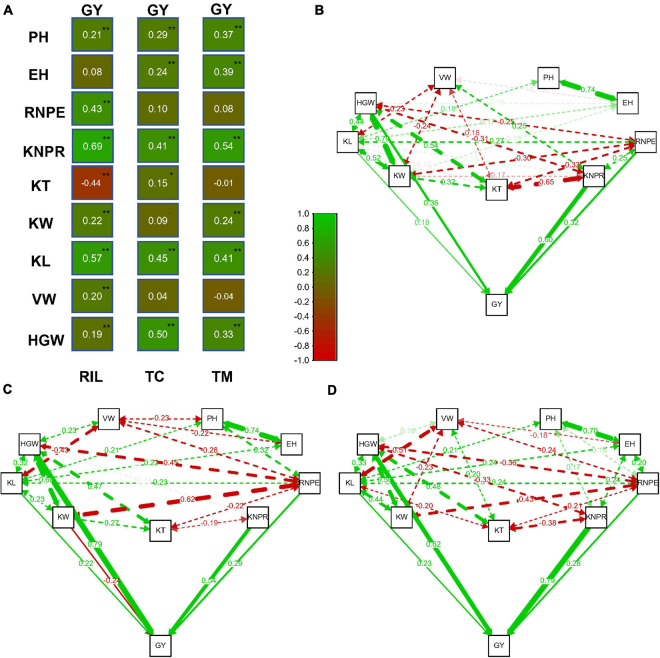
Correlation and path analysis of 10 traits in the recombinant inbred line population developed by Ye478 × Qi319 (RIL), Chang7-2 × RIL (TC), and Mo17 × RIL (TM) populations. **(A)** Correlation coefficients between grain yield per plant (GY) and the other traits. **(B)** Correlation and path coefficients between GY and the other traits in the RIL population, **(C)** in the TC population, and **(D)** in the TM population. The lines toward GY are the path coefficients and the other lines among the traits are correlation coefficients. Only coefficients larger than 0.14 (*p* = 0.01, *n* = 339) are displayed. **, significance at 0.01 level; *, significance at 0.05 level; PH, plant height; EH, ear height; RNPE, row number per ear; KNPR, kernel number per row; KT, kernel thickness; KW, kernel width; KL, kernel length; VW, volume weight; HGW, hundred grain weight; GY, grain yield per plant.

Relatively high correlations between traits were observed in the three populations (Figure 2A). The correlation between traits KNPR and GY was *r* = 0.69 (*p* < 0.01) and the correlation between KL and GY was 0.57 (*p* < 0.01) in the RIL population, which were the highest among the correlations between GY and the other traits. In the TC population, the highest correlations of GY occured between GY and HGW (*r* = 0.50, *p* < 0.01) and between GY and KL (*r* = 0.45, *p* < 0.01). In the TM population, the highest correlations were between GY and KNPR (*r* = 0.54, *p* < 0.01) and between GY and KL (*r* = 0.41, *p* < 0.01).

It is difficult to determine which trait contributes the most to the variation of grain yield only through correlation analysis between GY and the other traits. We therefore next performed a path analysis of all traits with GY (Figures 2B–D). By taking GY as the response variable and all other traits as independent variables, we estimated the path coefficients for every trait. In the RIL population, the highest path coefficients occurred for KNPR (0.60) and for HGW (0.36). The trait HGW had the highest path coefficient (0.79), followed by KNPR (0.54) in the TC population. In the TM population, the highest path coefficient was 0.79 for KNPR, followed by 0.62 for HGW. In summary, KNPR and HGW contributed most to the variation of grain yield.

### Main Effect Quantitative Trait Loci Mapping in the Recombinant Inbred Line, TC, and TM Populations

A high-density genetic map was constructed using 4,141 bins, covering 2669.49 cM of the maize genome (Supplementary Table 3 and Supplementary Figure 2). The average density of the marker map was 0.64 cM/bin in the whole genome, enabling a high resolution for QTL mapping.

To dissect the genetic architecture of the 10 traits, we first examined the additive model with the additive polygenic effect plus the additive-by-additive polygenic effect to control the genomic background (Supplementary Table 4). The additive (narrow-sense) heritability in the RIL population ranged from 0.25 for VW to 0.69 for PH. In the TC population, it ranged from 0.31 for VW to 0.70 for RNPE and in the TM population, it ranged from 0.38 for VW to 0.72 for EH. Generally, the proportion of phenotypic variance explained by the additive effects was greater than that explained by the additive-by-additive effects for all 10 traits. We also found that the proportion of variance explained by the additive-by-additive effects for the traits RNPE, KT, KW, KL, and GY was larger in the RIL population than the corresponding proportion in the TC and TM populations (Supplementary Table 4), illustrating that further studies are needed to understand the non-additive genetic architecture of these traits.

**FIGURE 3:**
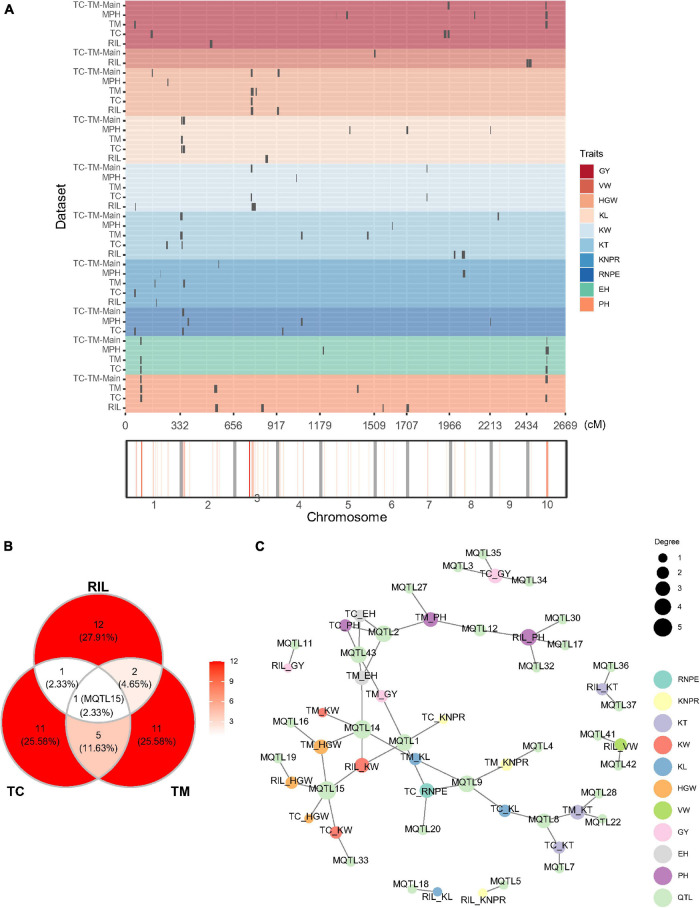
Quantitative trait loci (QTL) distribution and pleiotropic QTL detected in the recombinant inbred line population developed by Ye478 × Qi319 (RIL), Chang7-2 × RIL (TC), and Mo17 × RIL (TM) populations. **(A)** QTL distribution and hotspots in the whole genome shown for RIL, TC, and TM populations. TC-TM-Main is the mapping results for the additive and dominance effects of QTL from the pooled population of TC and TM. MPH represents the result of dominance QTL mapping for MPH. **(B)** Venn diagram showing the numbers of overlapping QTL between the RIL, TC, and TM populations. **(C)** Trait-QTL network for the 10 traits and QTL identified in the RIL, TC, and TM populations. The connections between traits and QTL are linked if a QTL was identified for the respective trait. PH, plant height; EH, ear height; RNPE, row number per ear; KNPR, kernel number per row; KT, kernel thickness; KW, kernel width; KL, kernel length; VW, volume weight; HGW, hundred grain weight; GY, grain yield per plant.

We also mapped QTL for the 10 traits in the RIL, TC, and TM populations, respectively (Figure 3A and Supplementary Table 5). In the RIL population, a total of 16 QTL were identified on eight chromosomes and five superior alleles were from the Ye478 parental line over the 10 traits. In the TC population, a total of 18 QTL were identified, among which 10 superior alleles came from the Ye478 parent. 19 QTL were identified in the TM population, among which eight superior alleles originated from the Ye478 parent. Three common QTL were jointly identified in the RIL and TM populations, two QTL were shared by the RIL and TC populations, and six QTL were jointly detected in the TC and TM populations (Figure 3B). One QTL (MQTL15) located in the interval from 162.93 to 172.23 Mb on chromosome 3 was shared among all three populations. This QTL was associated with KW in the RIL and the TC population and with HGW in all three populations (Figure 3C). A few other QTL also showed pleiotropic effects. For example, MQTL9 located between 1.71 and 4.67 Mb on chromosome 2 was associated with RNPE and KL in the TC population and with KNPR and KL in the TM population.

In the above QTL mapping results, low overlapping ratios among TC, TM and RIL populations were observed. In addition, in phenotype, the top 10 lines in the TC population did not match those identified in the TM population or vice versa (Supplementary Figures 3A,B). The two genetic phenomenons suggested that non-additive effects were important for hybrid performance. In this case, it is interesting to further dissect the hybrid performance to mine dominance and epistatic QTL.

### Multiple Variance Components Dissection and Main Effect Quantitative Trait Loci Mapping for Hybrid Performance and Midparent Heterosis in the TC-TM Population

We dissected the contribution of all five variance components (additive, dominance, and three epistatic polygenic variances) by Bayesian generalized linear regression (Pérez and De Los Campos, 2014) based on the hybrid performance and MPH in the TC-TM population. The results for the hybrid performance showed that additive-by-additive was the most important polygenic effect for the traits PH, EH, and KT, additive-by-dominance was predominant for VW and dominance was the most important polygenic effect for the remaining traits (Supplementary Table 6). For the analysis of MPH, the additive-by-dominance variance contributed the most for traits KT, KW, VW, and HGW, while the dominance variance contributed the most for the other six traits (Supplementary Table 7). Different proportions of dominant variances among 10 traits showed the complexity of heterosis.

**FIGURE 4:**
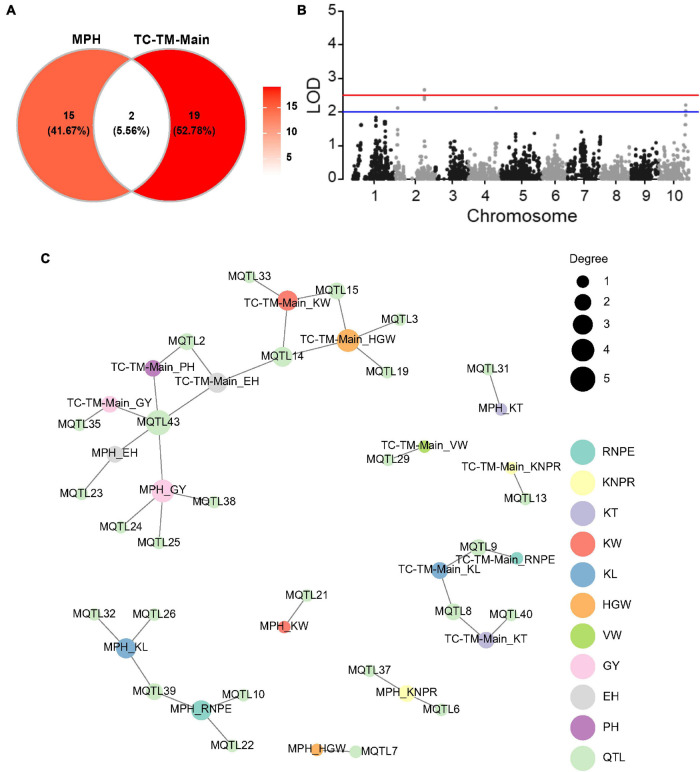
Results of the main effect quantitative trait loci (QTL) mapping in the pooled Chang7-2 × RIL (TC)-Mo17 × RIL (TM) population and dominance effect QTL mapping for midparent heterosis (MPH). **(A)** Venn diagram showing the numbers of pleiotropic QTL overlapping between TC-TM-Main and MPH. TC-TM-Main is the mapping results for the additive and dominance effects of QTL from the pooled population of TC and TM. MPH represents the result of dominance QTL mapping for midparent heterosis. **(B)** Dominance QTL identified for kernel number per row in the pooled TC-TM population. **(C)** Trait-QTL network for 10 traits and QTL identified in the TC-TM-Main and MPH datasets. The connections between traits and QTL are linked if a QTL was identified for this trait. PH, plant height; EH, ear height; RNPE, row number per ear; KNPR, kernel number per row; KT, kernel thickness; KW, kernel width; KL, kernel length; VW, volume weight; HGW, hundred grain weight; GY, grain yield per plant; LOD, log of odds.

We implemented a mixed model to test the main (additive and dominance) effects of a specific marker for both the hybrid performance and the MPH for all traits in the pooled TC-TM population. A total of 21 main effect QTL were identified for the 10 traits for hybrid performance (Supplementary Table 5 and Figure 4A). Among them, one had a significant dominance effect for KNPR and was located in the interval 210.29–211.57 Mb on chromosome 2 (Figure 4B and Supplementary Figure 4). For the other 20 QTL, the additive and dominance effects were confounded due to the fact that there were only two genotypes per locus (Supplementary Figure 4). Moreover, a total of 17 dominance QTL were detected for MPH for the 10 traits (Figure 4C and Supplementary Table 5). Interestingly, only two detected QTL were in common between MPH and hybrid performance (Figure 4A). The pleiotropic QTL MQTL43 located in the interval around 80.08–112.87 Mb on chromosome 10 was associated with EH and GY in the MPH dataset and with PH, EH and GY in the TC-TM-Main dataset (Supplementary Table 5 and Figure 4C). The lack of common QTL between MPH and hybrid performance implies that the two phenomena might have different genetic architectures, consistent with the results of the variance component analysis.

### Epistasis Plays an Important Role in Hybrid Performance

For hybrid performance in the TC-TM population, we scanned the entire genome to identify significant epistasis loci for the 10 traits and 197, 176, 131, and 112 significant epistatic pairs of loci were identified for additive-by-additive, additive-by-dominance, dominance-by-additive and dominance-by-dominance effects, respectively (Supplementary Table 8). The number of significant locus pairs varied across traits and the proportion of explained variance of an epistatic interaction ranged from 3.46 to 4.52%. For grain yield, only one significant additive-by-dominance QTL were detected. We observed the phenomenon of a continuous region interacting with another locus in the genome. For example, for additive-by-additive mapping, the interaction between a cluster of adjacent SNPs on chromosome 8 (Chr8_180048590, Chr8_180913576, Chr8_181023046, and Chr8_180032314) and a locus on chromosome 6 (Chr6_166754537) was significantly associated with PH (Supplementary Table 8).

**FIGURE 5:**
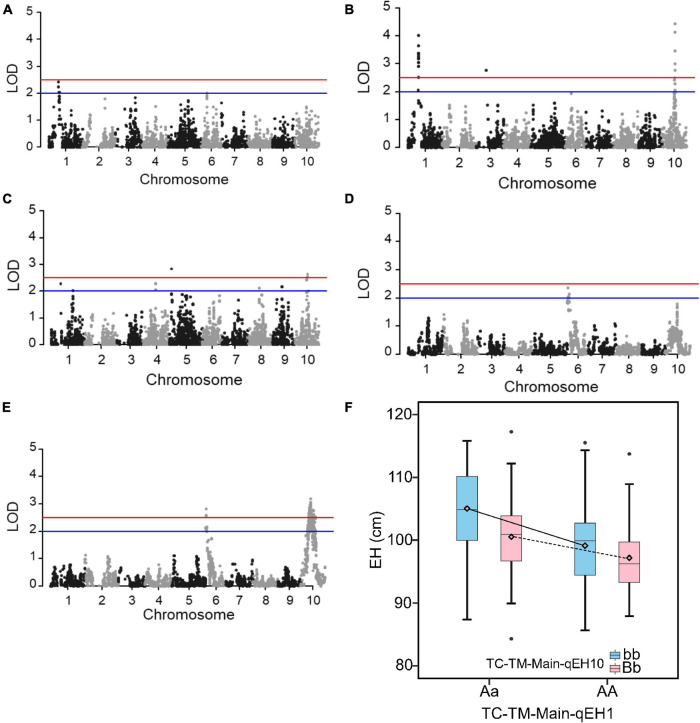
Quantitative trait loci (QTL) mapping results for the trait ear height. **(A)** QTL mapping in the recombinant inbred line population developed by Ye478 × Qi319 (RIL) population. **(B)** In the pooled Chang7-2 × RIL (TC)-Mo17 × RIL (TM) population, and **(C)** dominance QTL mapping for midparent heterosis. **(D)** Test for epistasis between the QTL *TC-TM-Main-qEH1* (peak single nucleotide polymorphisms is Chr1_131115160) on chromosome 1 and the other 4,140 markers in the RIL population. **(E)** Test for epistasis between the QTL *TC-TM-Main-qEH1* (peak single nucleotide polymorphisms is Chr1_131115160) on chromosome 1 and the other 4,140 markers in the pooled TC-TM population. The red horizontal line indicates the significance threshold used for QTL detection and the blue line is the threshold to identify the loci for the favorable QTL analysis. **(F)** The interactions between different genotypes of QTL *TC-TM-Main-qEH1* (Chr1_116118501; Aa, AA) and different genotypes of QTL *TC-TM-Main-qEH10* (Chr10_91890676; bb, Bb). The diamond indicates the mean value of different genotypes. LOD, log of odds.

EH had a more simple genetic architecture compared to GY and the variation of MPH for EH was also higher (Supplementary Table 1). We therefore used the trait EH as an example to investigate the epistatic effects in the RIL and the two hybrid populations. In the RIL population, no QTL was identified (Figure 5A). However, in the pooled TC-TM population, two main effect QTL for hybrid performance were identified on chromosomes 1 (*TC-TM-Main-qEH1* represented by the peak SNP Chr1_131115160) and 10 (*TC-TM-Main-qEH10* represented by the peak SNP Chr10_91890676) (Figure 5B). For MPH, however, only the *MPH-qEH10* QTL had a significant dominance effect as well as several additional small-effect QTL (Figure 5C). We further tested the additive-by-additive interactions between *TC-TM-Main-qEH1* and all other SNPs (4,140 in total). None of the tested effects were significant in the RIL population (Figure 5D). However, several significant interactions were identified in the pooled TC-TM population (Figure 5E). Further analysis confirmed the interaction between the two loci *TC-TM-Main-qEH1* and *TC-TM-Main-qEH10* in the pooled TC-TM population (Figure 5F). The specific type of epistatic effect between the two loci in the TC-TM population could not be determined because there were only two different genotypes at each locus. However, as we observed that the additive-by-additive effect was not significant between these two loci in the RIL population, we concluded that it is likely the additive-by-dominance or dominance-by-dominance effects that led to the detection of this epistatic QTL in the hybrid population but not in the RIL population.

### Correlation Between the Number of Favorable Quantitative Trait Loci and Hybrid Performance

We chose a slightly lower significance threshold of LOD = 2.0 to obtain more loci for this analysis, which yielded four and six significant QTL for GY in the TC and TM population, respectively. If the performance of heterozygous genotypes was better than that of homozygous genotypes at one QTL, it was called a heterozygous favorable QTL; otherwise, it was called a homozygous favorable QTL. The correlations between the number of favorable QTL and the hybrid performance were calculated for all 10 traits (Supplementary Table 9). The correlations between the hybrid performance and the number of favorable homozygous QTL (*r*_1_), the number of favorable heterozygous QTL (*r*_2_) and the total number of favorable QTL (*r*_3_) varied across traits, but were significant for most of traits in both the TC and TM populations.

**FIGURE 6:**
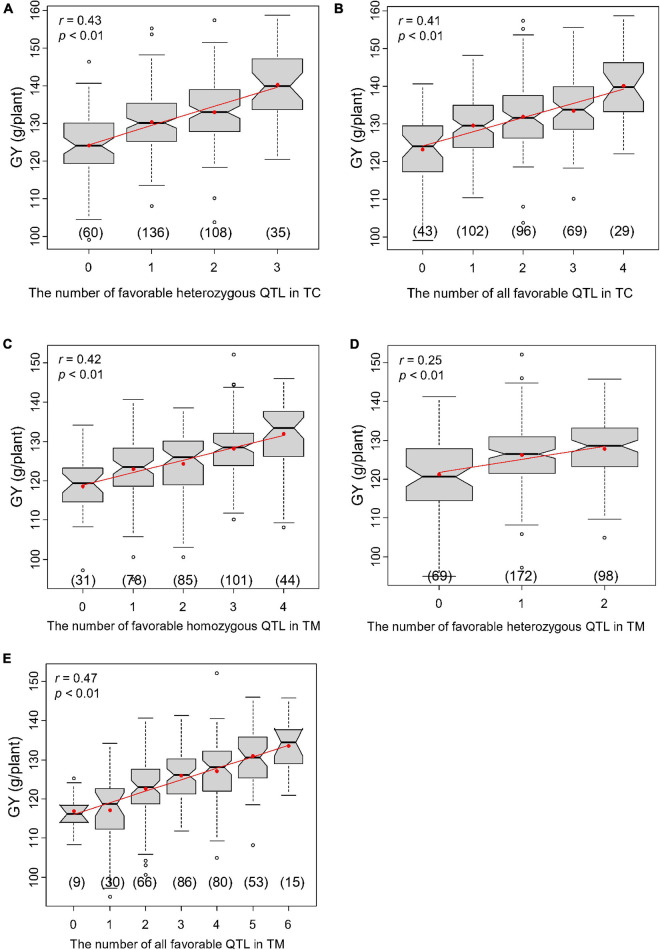
Correlations between the number of favorable quantitative trait loci (QTL) and grain yield per plant (GY). **(A)** Correlation between the number of all favorable QTL and GY in Chang7-2 × RIL (TC) population and **(B)** between the number of favorable heterozygous QTL and GY in TC population. **(C)** Correlation between the number of all favorable QTL and GY in Mo17 × RIL (TM) population and **(D)** between the number of favorable heterozygous QTL and GY in TM population. **(E)** Correlation between the number of favorable homozygous QTL and GY in TM population.

In the TC population, three of the four QTL for GY were heterozygous favorable QTL and *r*_1_, *r*_2_, and *r*_3_ were 0.16 (Supplementary Table 9), 0.43 (Figure 6A) and 0.41 (Figure 6B), respectively. In the TM population, only two of the six detected QTL were heterozygous favorable QTL and *r*_1_, *r*_2_, and *r*_3_ were 0.42 (Figure 6C), 0.25 (Figure 6D), and 0.47 (Figure 6E), respectively. These results illustrate that superior hybrids can be selected by combining favorable alleles at significant loci.

### Genomic Selection Accuracy in Different Breeding Schemes

**FIGURE 7:**
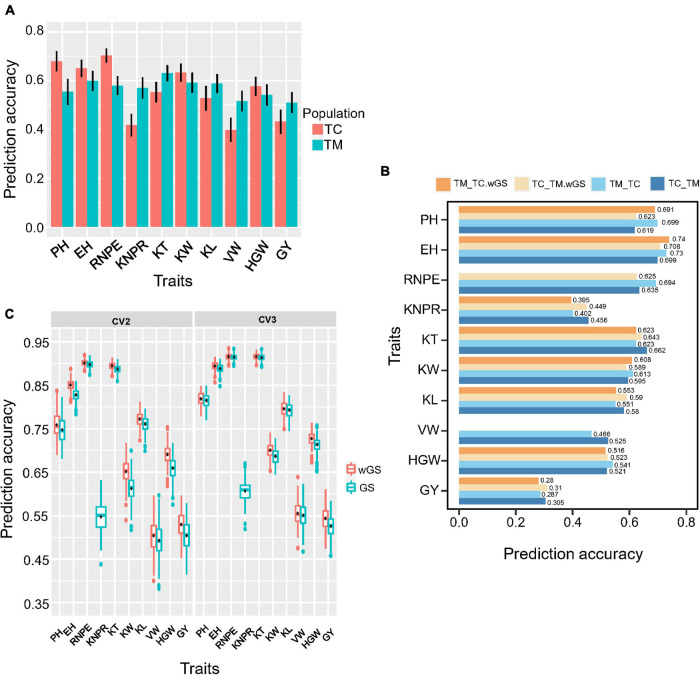
Genomic prediction accuracy of different cross-validation strategies. **(A)** Prediction accuracy within Chang7-2 × RIL (TC) and Mo17 × RIL (TM) population. RIL, the recombinant inbred line population developed by Ye478 × Qi319. **(B)** Prediction accuracy of 10 traits of cross-validation strategy 1 (CV1). TC_TM represents TM predicted by the TC population; TM_TC represents TC predicted by the TM population. **(C)** Prediction accuracy of 10 traits of cross-validation strategy 2 (CV2) and cross-validation strategy 3 (CV3). wGS is a weighted genomic selection that incorporates the peak single nucleotide polymorphisms (SNP) of potential target quantitative trait loci as fixed effects. PH, plant height; EH, ear height; RNPE, row number per ear; KNPR, kernel number per row; KT, kernel thickness; KW, kernel width; KL, kernel length; VW, volume weight; HGW, hundred grain weight; GY, grain yield per plant.

For genomic selection within populations, the prediction accuracy ranged from 0.70 for RNPE to 0.40 for VW in TC and ranged from 0.63 for KT to 0.51 for GY in TM population (Figure 7A). Generally, traits with a low heritability usually had a low prediction accuracy, like VW in TC and GY in TM population.

The three cross-validation schemes were shown in Supplementary Figure 1. For strategy 1 (CV1), when using the TC population to predict the TM population (TC_TM), the prediction accuracy ranged from 0.305 for GY to 0.699 for EH (Figure 7B). Conversely, the prediction accuracy ranged from 0.287 for GY to 0.73 for EH when the TM population was used to predict the TC population (TM_TC). When the significant QTL identified in the training population were included as fixed effects in the prediction model, this did not result in an improvement of the prediction accuracy for most of the traits. Only a few traits, e.g., EH and KL, showed a slight improvement. For some traits, e.g., KNPR and KT, the prediction accuracy even decreased.

For the second cross-validation strategy (CV2), the lowest prediction accuracy was 0.49 for VW and the highest prediction accuracy was 0.90 for RNPE. For the third cross-validation strategy (CV3), the lowest prediction accuracy was 0.53 for GY and the highest prediction accuracy was 0.92 for KT and RNPE (Figure 7C). The results also showed that the prediction accuracy of CV3 was higher than within population scheme and CV1, CV2, regardless of whether GS or wGS was applied. The wGS taking potential QTL as fixed had a higher prediction accuracy than GS in both CV2 and CV3 (Figure 7C).

## Discussion

### Hundred Grain Weight and Kernel Number per Row Significantly Contribute to the Variation of Grain Yield

Grain yield is a complex trait, affected by many genetic and non-genetic factors. The three traits that were found to mainly contribute to GY are HGW, RNPE, and KNPR. In earlier studies, the focus has been placed on correlation analysis between traits. In general, moderate to high correlations were observed between GY and many other traits (Cui et al., 2016; Li et al., 2020, 2021). However, it is difficult to determine which trait contributes the most to grain yield. Path analysis is an alternative approach that allows examining the relative importance of a component trait to the variation of the target trait. Our results revealed that regardless of whether the population was the RIL population or the hybrid population, HGW and KNPR had the highest path coefficients thus, contributed the most to the variation of GY (Figures 2B–D). Consequently, HGW and KNPR are promising indirect traits to improve GY in hybrid breeding in maize.

### Identification of Quantitative Trait Loci Using an Additive Genetic Model

Previous studies in the underlying populations (RIL, TC, and TM) focused on additive genetic models, where the genotypes and phenotypes of the RIL population were used to map QTL by treating the GCA as traits (Zhou et al., 2018; Lu et al., 2020). In this study, the genotypes and phenotypes of two hybrid populations were used to detect significant main (additive and dominance) effects and epistatic QTL. Only two common QTL were identified between the RIL and the TC hybrid population, and three common QTL were identified between the RIL and the TM hybrid population (Figure 3B). These results indicated that the non-additive genetic effect played an important role, which meant that a line with a moderate value of GY can still yield a high GY when crossing with testers (Supplementary Figure 3). And an additive model was not enough to explain heterosis.

### Non-additive Polygenic Effects Play an Important Role in Hybrid Performance

The proportion of phenotypic variance explained by the additive-by-additive effects in the RIL population was higher for most traits than that in the TC and TM populations (Supplementary Table 4). And the unparallel relationship between RILs and hybrid populations (Supplementary Figure 3) inspired further exploration of the genetic basis in the combined TC-TM hybrid population using a model integrating non-additive polygenic effects. Based on the estimated variance components, we conclude that the prominent gene action varies across traits (Supplementary Table 6). For example, the prominent variance was the additive-by-additive component for the trait PH, but for GY the prominent variance was the dominance component. This presents a considerable challenge for selecting elite single-cross hybrids and for uncovering the importance of non-additive genetic effects because additive variance is not a prominent factor to control the variation of any of the investigated traits.

### Identification of Quantitative Trait Loci Using a Non-additive Model

For genetic dissection of the hybrid performance, Xu (2013) proposed a new mixed model method for QTL mapping by incorporating multiple polygenic covariance structures, which consist of the additive, dominance, and epistatic variance components. In theory, each particular effect could be tested in a model by controlling all other genetic effects as background. Similarly, a quantitative genetic framework was proposed for the genetic dissection of MPH (Jiang et al., 2017). The above two linear mixed models are very similar in the polygenic background control. The main difference is the response variable, in the former it is the performance of the hybrid (Xu, 2013) and in the latter the MPH (Jiang et al., 2017). It should be noted that the hybrid performance could not be simply replaced by MPH with just the removal of the additive effect from the linear mixed model (Jiang et al., 2017). In our study, the GBS technology only covered about 0.07-fold of the genome in our populations. The two tester lines had the same genotype at 95% of the loci, which resulted in the pooled TC-TM population having just two genotypes at 95% of loci. Two genotypes per locus mimick a backcross population so that the additive effects are confounded with the dominance effects, which explains why the TC-TM-Add model was nearly the same as the TC-TM-Dom model (with one exception for a dominance QTL) (Supplementary Figure 4). Regardless of the high similarity between the hybrid populations and the hypothetical BC population, the hybrid populations still had advantages. In a dominance test for MPH, 17 significant dominance loci were detected (Figure 4A) and for hybrid performance, a set of significant epistatic loci was identified (Supplementary Table 8).

### Improve Prediction Accuracy by Integrating Functional Markers

In genomic selection models like GBLUP or rrBLUP, all SNPs were treated equally or had the same distribution when treated as random. Actually, significant QTL contributed more to the variation of traits. In such a case, significant SNPs should be treated differently. In this study, those SNPs were included in the fixed effect in the GS model to explore whether prediction accuracy could be improved. For cross-validation scheme 1, just two of 10 traits, namely EH and KL showed slight improvement. We guess that TC and TM populations had different genetic backgrounds (Li et al., 2019) and those QTL made different effect within two different populations. So the QTL effect was estimated biasedly when only one tester population as training population. But for CV2 and CV3, the population used for QTL mapping consisted of lines from both TC and TM, in this case, improvements were observed for all traits harboring QTL (Figure 7C). By comparison, it showed that CV3 scheme had superiority over the within population scheme, CV1, and CV2, which inspired us that when there were some known functional QTL in a target population, a strategy treating known QTL as fixed effects with CV3 design was a better choice for genomic prediction.

### Relationship Between Midparent Heterosis and Hybrid Performance

Hybrid performance is the phenotypic value of the hybrid, which is the sum of the midparent heterosis and the midparent value. Hybrid performance is controlled by the additive, the dominance and all four epistatic polygenic effects, whereas MPH is not affected by the additive effect because the additive effect does not contribute to heterosis (Jiang et al., 2017). In this study, we confirmed the different genetic architecture of hybrid performance and MPH as both had only two QTL in common (Figure 4A), which is consistent with a previous study (Hua et al., 2003). Furthermore, the variance component ratios were also different between hybrid performance and MPH (Supplementary Tables 6, 7). In a wheat study, the midparent value showed a negative correlation with MPH but was positively correlated with the hybrid performance (Boeven et al., 2020). In our study, we observed the correlation between the hybrid performance and MPH was 0.77 (*p* < 0.01) (Supplementary Figure 5A), and a positive correlation of 0.23 (*p* < 0.01) between the hybrid performance and midparent value (Supplementary Figure 5B), while a negative correlation between midparent value and MPH for grain yield of −0.45 (*p* < 0.01) (Supplementary Figure 5C). And the path analysis also highlighted the superior contribution of MPH to hybrid performance in hybrid population (Supplementary Figure 5D). In plant hybrid breeding, we aim to select a single-cross hybrid with both high MPH and midparent value, but these seem to be contradictory goals considering the negative correlation. Consequently, hybrid breeding must balance the two and target hybrid performance to achieve high performing hybrids.

### Mechanisms of Midparent Heterosis and Hybrid Performance

Although dominant and additive effects couldn’t be separated and dominant degree couldn’t be estimated in this study, multiple variance components dissection provided possibility to assess the mechanism of heterosis and hybrid performance. Results showed the dominance contributed the highest proportion for MPH of most traits, especially for GY and KNPR (Supplementary Table 7). However, it was found that the epistasis (sum of additive-by-additive, additive-by-dominance, and dominance-by-dominance) contributes the highest proportion to hybrid performance of GY, PH, EH, and KNPR (Supplementary Table 6). The results were similar to a previous report in maize (Tang et al., 2010). A series of linear mixed models incorporating multiple polygenic covariance structures together with NCII population provide possibility to explore the genetic factors and mechanism of heterosis.

## Data Availability Statement

The original contributions presented in the study are included in the article/Supplementary Material, further inquiries can be directed to the corresponding authors.

## Author Contributions

WL, ML, and SX designed the study. ZZ collected the phenotypic data. DL, XiaL, GL, JL, and HW performed data analysis. DL drafted the manuscript. DL, XiaL, YJ, SC, TW, JR, XinL, SX, ML, and WL revised the manuscript. All authors read and approved the final manuscript.

## Conflict of Interest

The authors declare that the research was conducted in the absence of any commercial or financial relationships that could be construed as a potential conflict of interest.

## Publisher’s Note

All claims expressed in this article are solely those of the authors and do not necessarily represent those of their affiliated organizations, or those of the publisher, the editors and the reviewers. Any product that may be evaluated in this article, or claim that may be made by its manufacturer, is not guaranteed or endorsed by the publisher.

## Supplementary Material

Supplementary Figure 1 Three different cross-validation schemes. **(A)** Cross-validation strategy 1. **(B)** Cross-validation strategy 2. **(C)** Cross-validation strategy 3. RILs, the recombinant inbred lines developed by Ye478 × Qi319.

Supplementary Figure 2 Collinearity between the genetic and physical maps.

Supplementary Figure 3 Parallel maps of grain yield per plant (GY) in the recombinant inbred line population developed by Ye478 × Qi319 (RIL), Chang7-2 × RIL (TC), and Mo17 × RIL (TM) populations. **(A)** GY was ranked based on the TC population. **(B)** GY was ranked based on the TM population.

Supplementary Figure 4 Venn diagram showing the numbers of pleiotropic quantitative trait loci (QTL) overlapping among TC-TM-Add, TC-TM-Dom and MPH. TC-TM-Add represents the mapping results for the additive effects in the pooled population of Chang7-2 × RIL (TC) and Mo17 × RIL (TM). RIL, the recombinant inbred line population developed by Ye478 × Qi319; TC-TM-Dom represents the mapping results for the dominance effects in the pooled population of TC and TM; MPH represents the result of dominance QTL mapping for midparent heterosis.

Supplementary Figure 5 Correlations and path coefficients among hybrid performance, midparent heterosis (MPH) and midparent value. **(A)** Correlation between MPH and hybrid performance. **(B)** Correlation between midparent value and hybrid performance. **(C)** Correlation between midparent value and MPH. **(D)** The path coefficients among hybrid performance, MPH and midparent value.

Supplementary Table 1 Summary statistics for 10 traits for midparent heterosis in the Chang7-2 × RIL (TC), and Mo17 × RIL (TM) populations.

Supplementary Table 2 Variance of general combining ability (GCA) and specific combining ability (SCA) and their interaction with the environment.

Supplementary Table 3 Summary statistics for the genetic distances across 10 linkage groups of the maize genome.

Supplementary Table 4 Variance components and proportion of the phenotypic variance contributed by each variance component in the recombinant inbred line population developed by Ye478 × Qi319 (RIL), Chang7-2 × RIL (TC), and Mo17 × RIL (TM) populations, respectively.

Supplementary Table 5 QTL mapping results for 10 traits in the recombinant inbred line population developed by Ye478 × Qi319 (RIL), Chang7-2 × RIL (TC), Mo17 × RIL (TM) populations, the pooled TC-TM population and heterosis dataset.

Supplementary Table 6 Variance components and proportion of the phenotypic variance contributed by each variance component in the pooled Chang7-2 × RIL (TC)-Mo17 × RIL (TM) population.

Supplementary Table 7 Variance components and proportion of the phenotypic variance contributed by each variance component for midparent heterosis.

Supplementary Table 8 Significant epistatic paired loci for 10 traits identified from the pooled Chang7-2 × RIL (TC)-Mo17 × RIL (TM) population.

Supplementary Table 9 Correlations between the number of harbored favorable quantitative trait loci (QTL) and hybrid performance for 10 traits in Chang7-2 × RIL (TC) and Mo17 × RIL (TM) population, respectively.
