# Supplementary figures and images for: Genetic Dissection of Hybrid Performance and Heterosis for Yield-Related Traits in Maize
Source: Front Plant Sci. 2021 Nov 30;12:774478. doi: 10.3389/fpls.2021.774478 (PMC8670227; doi:10.3389/fpls.2021.774478)

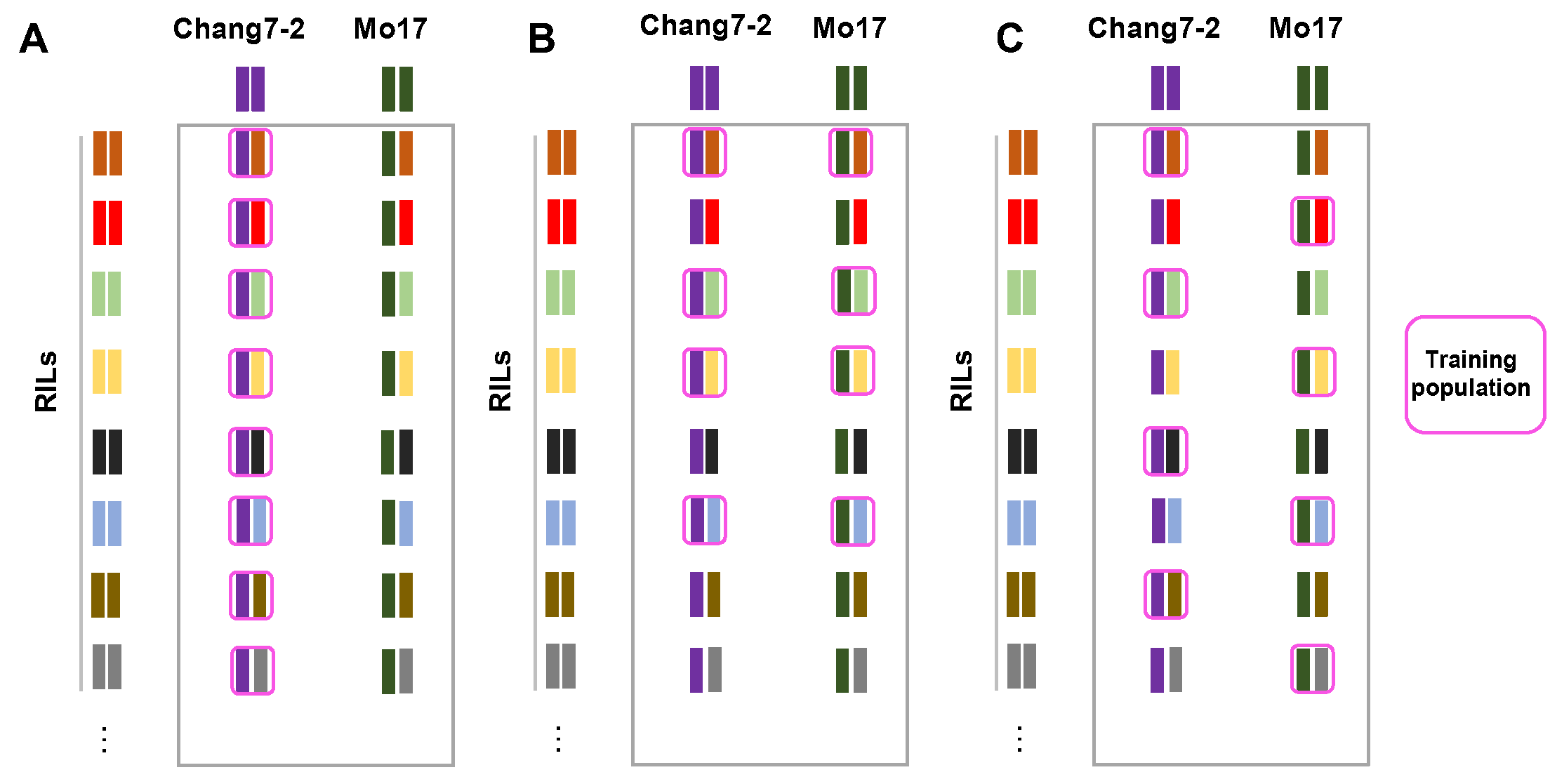

Supplement: Supplementary Figure 1 — Three different cross-validation schemes. (A) Cross-validation strategy 1. (B) Cross-validation strategy 2. (C) Cross-validation strategy 3. RILs, the recombinant inbred lines developed by Ye478 × Qi319. [file Image_1.TIFF]

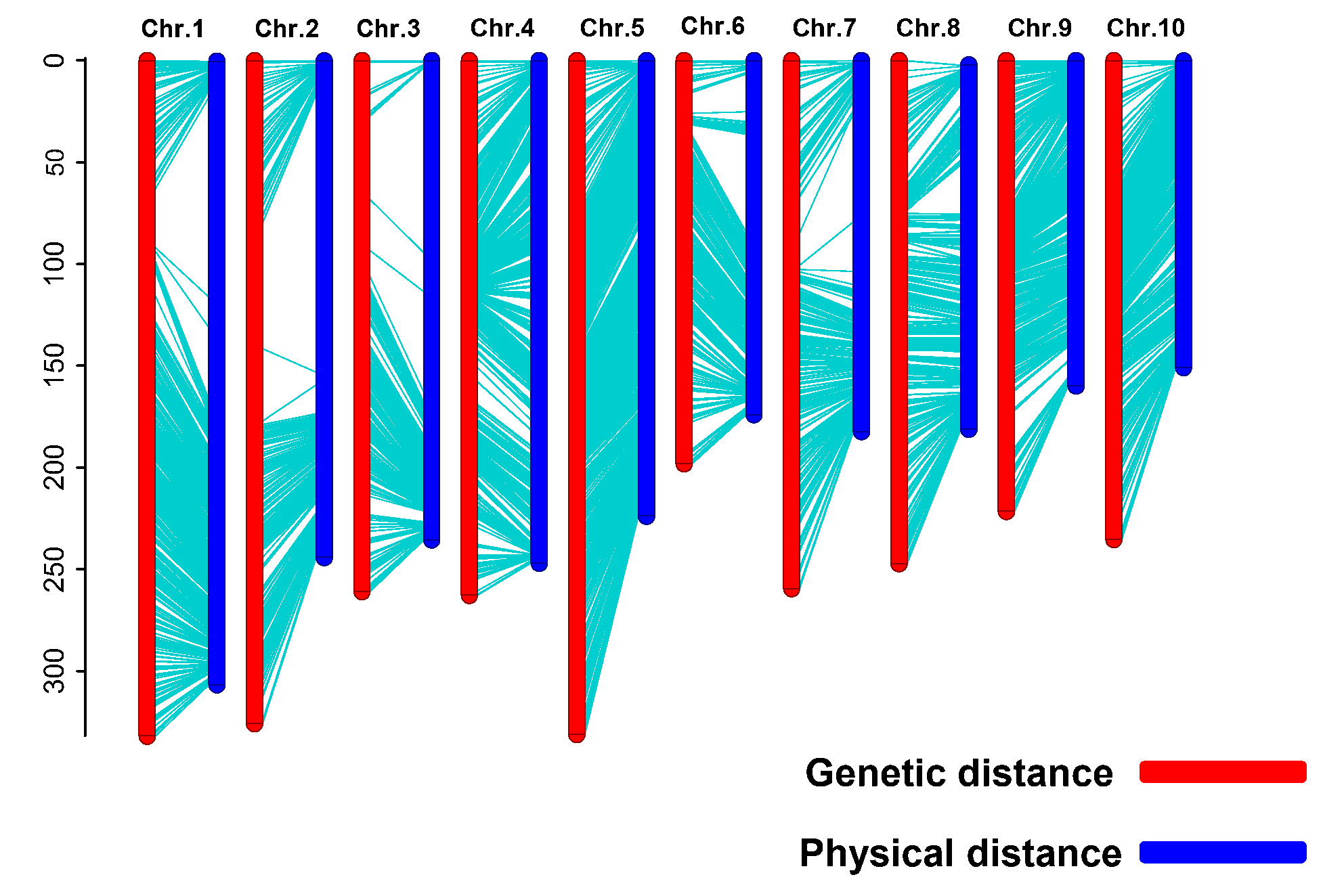

Supplement: Supplementary Figure 2 — Collinearity between the genetic and physical maps. [file Image_2.TIFF]

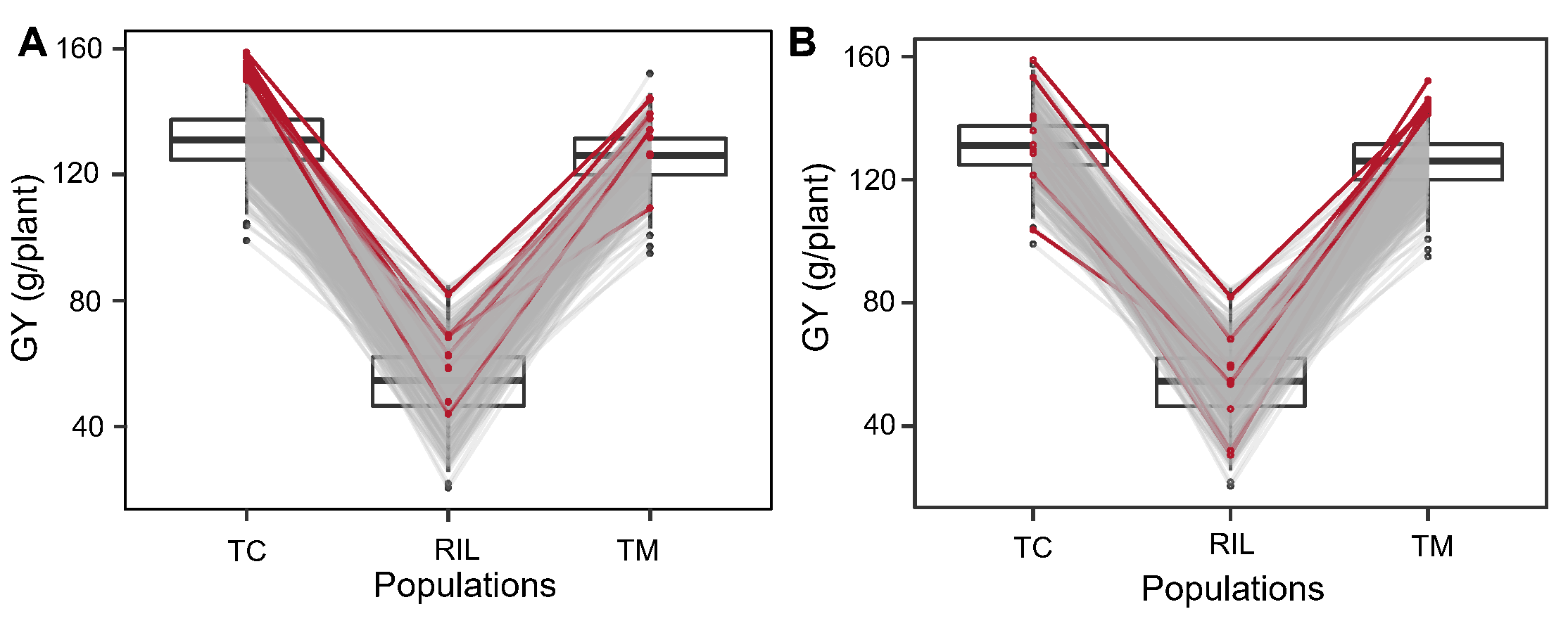

Supplement: Supplementary Figure 3 — Parallel maps of grain yield per plant (GY) in the recombinant inbred line population developed by Ye478 × Qi319 (RIL), Chang7-2 × RIL (TC), and Mo17 × RIL (TM) populations. (A) GY was ranked based on the TC population. (B) GY was ranked based on the TM population. [file Image_3.TIFF]

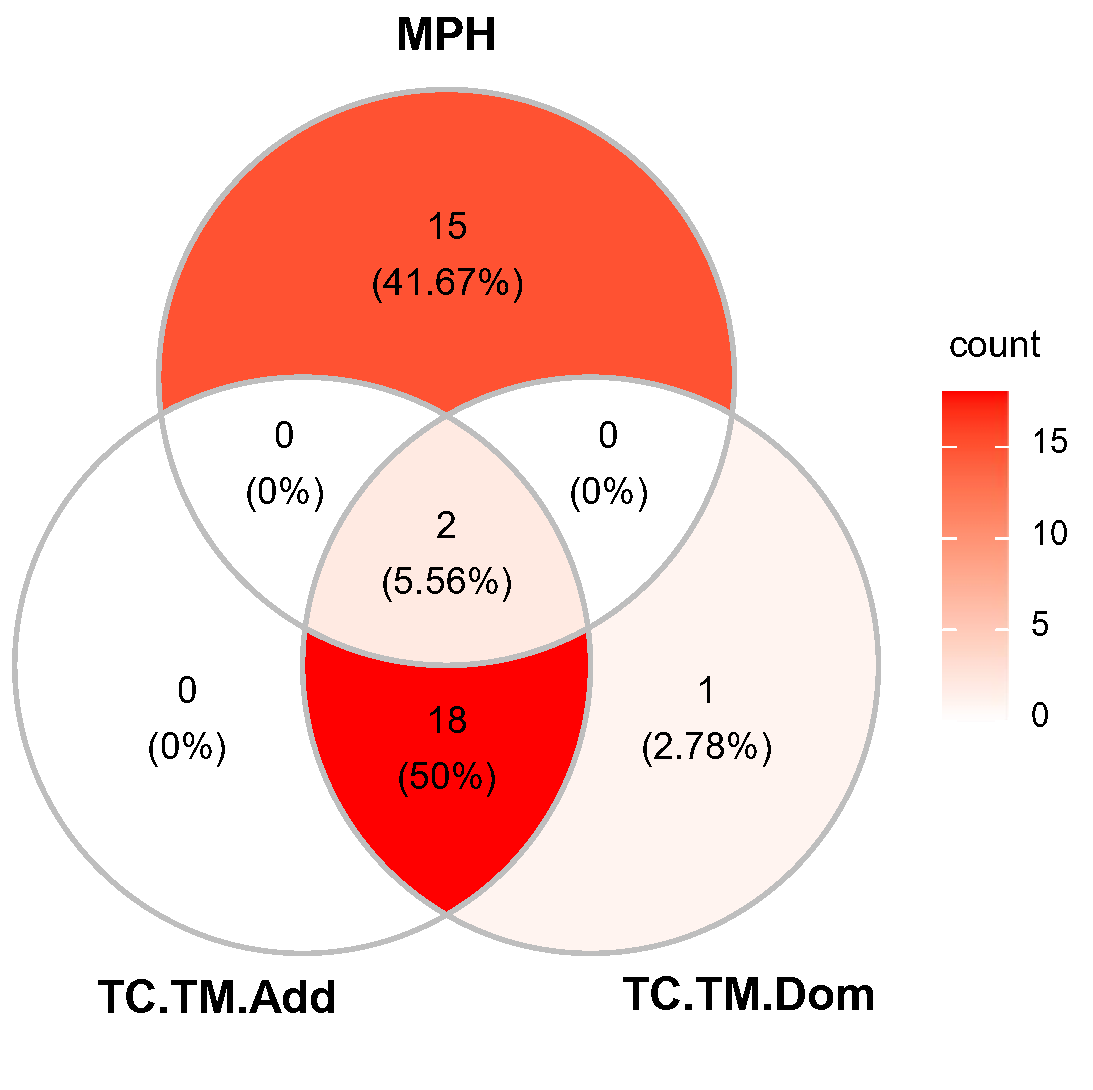

Supplement: Supplementary Figure 4 — Venn diagram showing the numbers of pleiotropic quantitative trait loci (QTL) overlapping among TC-TM-Add, TC-TM-Dom and MPH. TC-TM-Add represents the mapping results for the additive effects in the pooled population of Chang7-2 × RIL (TC) and Mo17 × RIL (TM). RIL, the recombinant inbred line population developed by Ye478 × Qi319; TC-TM-Dom represents the mapping results for the dominance effects in the pooled population of TC and TM; MPH represents the result of dominance QTL mapping for midparent heterosis. [file Image_4.TIFF]

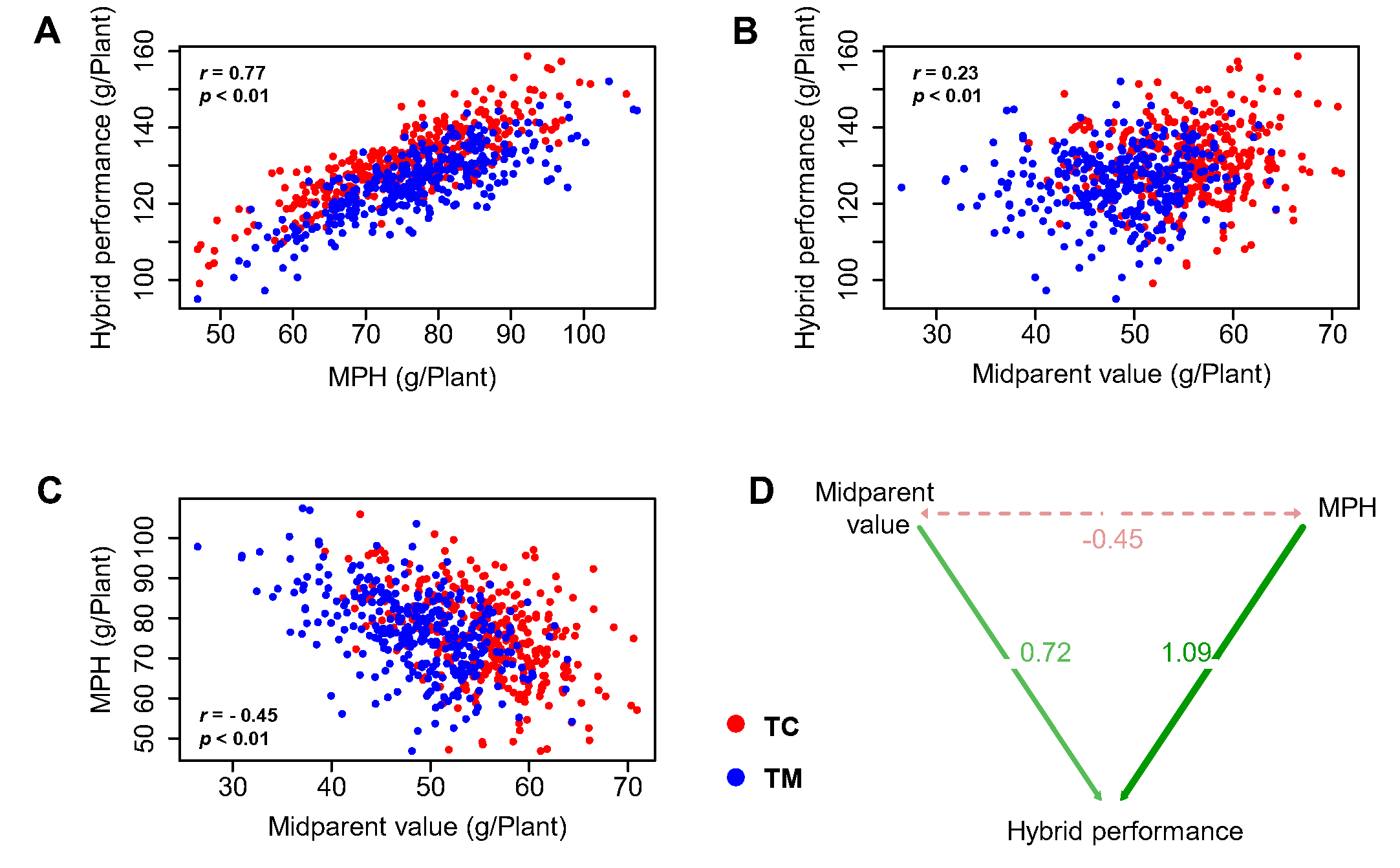

Supplement: Supplementary Figure 5 — Correlations and path coefficients among hybrid performance, midparent heterosis (MPH) and midparent value. (A) Correlation between MPH and hybrid performance. (B) Correlation between midparent value and hybrid performance. (C) Correlation between midparent value and MPH. (D) The path coefficients among hybrid performance, MPH and midparent value. [file Image_5.TIFF]
