# Supplementary material for: Genetic Dissection of Hybrid Performance and Heterosis for Yield-Related Traits in Maize
Source: Front Plant Sci. 2021 Nov 30;12:774478. doi: 10.3389/fpls.2021.774478 (PMC8670227; doi:10.3389/fpls.2021.774478)
Supplement: Supplementary Table 1 — Summary statistics for 10 traits for midparent heterosis in the Chang7-2 × RIL (TC), and Mo17 × RIL (TM) populations. [file Table_1.docx]

**Supplementary Table 1 |** Summary statistics for 10 traits for midparent heterosis in the Chang7-2 × RIL (TC), and Mo17 × RIL (TM) populations

|  | Traits | Min | Max | Mean | SD | CV  (%) | $\sigma_{G}^{2}$ | | $\sigma_{\varepsilon}^{2}$ | | N*_E_* | | | *H*^2^ | |  |
| --- | --- | --- | --- | --- | --- | --- | --- | --- | --- | --- | --- | --- | --- | --- | --- | --- |
| TC | PH | 42.99 | 88.04 | 63.26 | 7.23 | 11.44 | 40.07^**^ | | 37.00 | | | 4 | | 0.81 | |  |
|  | EH | 24.20 | 51.56 | 38.39 | 4.40 | 11.47 | 12.49^**^ | | 22.66 | | | 4 | | 0.69 | |  |
|  | RNPE | -0.50 | 3.37 | 1.23 | 0.59 | 47.63 | 0.23^**^ | | 0.40 | | | 4 | | 0.69 | |  |
|  | KNPR | 4.98 | 20.42 | 15.16 | 2.00 | 13.21 | 2.65^**^ | | 4.40 | | | 4 | | 0.71 | |  |
|  | KT | -13.06 | 6.98 | -5.94 | 1.85 | -31.09 | 2.33^**^ | | 3.26 | | | 4 | | 0.74 | |  |
|  | KW | 0.33 | 19.17 | 10.10 | 2.64 | 26.16 | 4.19^**^ | | 8.74 | | | 4 | | 0.66 | |  |
|  | KL | 9.92 | 34.90 | 22.85 | 3.84 | 16.80 | 9.79^**^ | | 16.71 | | | 4 | | 0.70 | |  |
|  | VW | -113.04 | 10.31 | -50.21 | 22.74 | -45.30 | 157.77^**^ | | 1143.21 | | | 4 | | 0.36 | |  |
|  | HGW | -0.28 | 10.62 | 4.20 | 1.74 | 41.53 | 1.76^**^ | | 5.09 | | | 5 | | 0.63 | |  |
|  | GY | 46.86 | 105.88 | 75.63 | 10.87 | 14.37 | | 71.80^**^ | | 173.98 | | | 5 | | 0.67 | |
| TM | PH | 48.77 | 96.77 | 67.78 | 6.69 | 9.87 | | 32.44^**^ | | 36.86 | | | 4 | | 0.78 | |
|  | EH | 18.19 | 49.56 | 31.44 | 4.38 | 13.91 | | 12.24^**^ | | 20.87 | | | 4 | | 0.70 | |
|  | RNPE | 0.51 | 3.16 | 1.86 | 0.41 | 22.29 | | 0.10^**^ | | 0.22 | | | 4 | | 0.65 | |
|  | KNPR | 5.78 | 20.24 | 14.77 | 2.37 | 16.03 | | 3.59^**^ | | 6.37 | | | 4 | | 0.69 | |
|  | KT | -10.68 | 6.11 | -2.90 | 2.26 | -77.92 | | 3.42^**^ | | 5.17 | | | 4 | | 0.73 | |
|  | KW | -2.17 | 12.27 | 4.08 | 2.21 | 54.33 | | 2.45^**^ | | 7.57 | | | 4 | | 0.56 | |
|  | KL | 9.40 | 26.18 | 17.02 | 3.12 | 18.32 | | 5.64^**^ | | 12.74 | | | 4 | | 0.64 | |
|  | VW | -74.20 | 68.77 | -10.14 | 24.26 | -239.11 | | 125.35^**^ | | 1008.65 | | | 3 | | 0.27 | |
|  | HGW | 0.11 | 12.79 | 4.83 | 1.61 | 33.35 | | 1.29^**^ | | 4.91 | | | 5 | | 0.57 | |
|  | GY | 46.84 | 107.39 | 76.86 | 10.00 | 13.02 | | 58.64^**^ | | 158.44 | | | 5 | | 0.65 | |

SD, standard deviation; CV, coefficient of variation; $\sigma_{G}^{2}$, genetic variance; $\sigma_{\varepsilon}^{2}$, error variance; N*_E_*, the number of environments; *H*^2^, broad-sense heritability; **, significance at 0.01 level. PH, plant height; EH, ear height; RNPE, row number per ear; KNPR, kernel number per row; KT, kernel thickness; KW, kernel width; KL, kernel length; VW, volume weight; HGW, hundred grain weight; GY, grain yield per plant.
