# Supplementary material for: Genetic Dissection of Hybrid Performance and Heterosis for Yield-Related Traits in Maize
Source: Front Plant Sci. 2021 Nov 30;12:774478. doi: 10.3389/fpls.2021.774478 (PMC8670227; doi:10.3389/fpls.2021.774478)
Supplement: Supplementary Table 2 — Variance of general combining ability (GCA) and specific combining ability (SCA) and their interaction with the environment. [file Table_2.DOCX]

**Supplementary Table 2** | Variance of general combining ability (GCA) and specific combining ability (SCA) and their interaction with the environment

| Traits | $\sigma_{E}^{2}$ | $\sigma_{{GCA}_{RIL}}^{2}$ | $\sigma_{{GCA}_{Tester}}^{2}$ | $\sigma_{SCA}^{2}$ | $\sigma_{{GCA}_{RIL}*E}^{2}$ | $\sigma_{{GCA}_{Tester}*E}^{2}$ | $\sigma_{SCA*E}^{2}$ | $\sigma_{\varepsilon}^{2}$ | SCA/GCA |
| --- | --- | --- | --- | --- | --- | --- | --- | --- | --- |
| PH | 167.31 | 57.58 | 69.98 | 13.75 | 7.72 | 6.28 | 0.00 | 23.91 | 0.11 |
| EH | 78.21 | 28.85 | 79.11 | 5.73 | 2.77 | 0.24 | 0.00 | 16.77 | 0.05 |
| RNPE | 0.06 | 0.22 | 1.43 | 0.10 | 0.01 | 0.03 | 0.00 | 0.23 | 0.06 |
| KNPR | 4.50 | 1.75 | 0.72 | 1.02 | 0.68 | 0.25 | 0.00 | 3.51 | 0.41 |
| KT | 0.18 | 2.89 | 14.77 | 0.68 | 0.10 | 2.69 | 0.00 | 2.19 | 0.04 |
| KW | 1.32 | 4.78 | 0.03 | 1.76 | 0.29 | 0.22 | 0.00 | 5.76 | 0.37 |
| KL | 20.42 | 6.36 | 11.93 | 2.61 | 0.94 | 10.87 | 0.00 | 10.87 | 0.14 |
| VW | 2692.67 | 174.80 | 1.03 | 64.63 | 55.28 | 127.54 | 0.01 | 698.74 | 0.37 |
| HGW | 0.65 | 1.02 | 0.66 | 0.70 | 0.55 | 1.15 | 0.00 | 3.93 | 0.42 |
| GY | 16.62 | 16.93 | 10.29 | 21.09 | 15.60 | 26.01 | 0.16 | 146.02 | 0.77 |

SCA/GCA was calculated by $\sigma_{SCA}^{2}$/($\sigma_{{GCA}_{RIL}}^{2}$+$\sigma_{{GCA}_{Tester}}^{2}$). $\sigma_{E}^{2}$, environment variance; $\sigma_{{GCA}_{RIL}}^{2}$, variance of GCA of RIL; $\sigma_{{GCA}_{Tester}}^{2}$, variance of GCA of tester; $\sigma_{SCA}^{2}$, variance of specific combining ability; $\sigma_{{GCA}_{RIL}*E}^{2}$, $\sigma_{{GCA}_{Tester}*E}^{2}$, $\sigma_{SCA*E}^{2}$ are the variance of the interactions between environment and GCA of RIL, GCA of tester, specific combining ability, respectively; $\sigma_{\varepsilon}^{2}$, error variance. PH, plant height; EH, ear height; RNPE, row number per ear; KNPR, kernel number per row; KT, kernel thickness; KW, kernel width; KL, kernel length; VW, volume weight; HGW, hundred grain weight; GY, grain yield per plant.
