# Supplementary material for: Genetic Dissection of Hybrid Performance and Heterosis for Yield-Related Traits in Maize
Source: Front Plant Sci. 2021 Nov 30;12:774478. doi: 10.3389/fpls.2021.774478 (PMC8670227; doi:10.3389/fpls.2021.774478)
Supplement: Supplementary Table 3 — Summary statistics for the genetic distances across 10 linkage groups of the maize genome. [file Table_3.DOCX]

**Supplementary Table 3 |** Summary statistics for the genetic distances across 10 linkage groups of the maize genome

| Linkage Group | Length(cM) | Bin Number | Density (cM/bin) |
| --- | --- | --- | --- |
| 1 | 331.80 | 487 | 0.68 |
| 2 | 324.18 | 267 | 1.21 |
| 3 | 260.85 | 262 | 1.00 |
| 4 | 262.62 | 549 | 0.48 |
| 5 | 329.41 | 700 | 0.47 |
| 6 | 197.89 | 312 | 0.63 |
| 7 | 259.36 | 277 | 0.94 |
| 8 | 246.74 | 403 | 0.61 |
| 9 | 221.39 | 426 | 0.52 |
| 10 | 235.25 | 458 | 0.51 |
| Total | 2669.49 | 4141 | 0.64 |
