# Supplementary material for: Genetic Dissection of Hybrid Performance and Heterosis for Yield-Related Traits in Maize
Source: Front Plant Sci. 2021 Nov 30;12:774478. doi: 10.3389/fpls.2021.774478 (PMC8670227; doi:10.3389/fpls.2021.774478)
Supplement: Supplementary Table 4 — Variance components and proportion of the phenotypic variance contributed by each variance component in the recombinant inbred line population developed by Ye478 × Qi319 (RIL), Chang7-2 × RIL (TC), and Mo17 × RIL (TM) populations, respectively. [file Table_4.docx]

**Supplementary Table 4 |** Variance components and proportion of the phenotypic variance contributed by each variance component in the recombinant inbred line population developed by Ye478 × Qi319 (RIL), Chang7-2 × RIL (TC), and Mo17 × RIL (TM) populations, respectively

| Population | Traits | $\sigma_{a}^{2}$ | $\sigma_{aa}^{2}$ | $\sigma_{\varepsilon}^{2}$ | $h_{a}^{2}$ | $h_{aa}^{2}$ | $h^{2}$ |
| --- | --- | --- | --- | --- | --- | --- | --- |
| RIL | PH | 211.11 | 35.57 | 59.53 | 0.69 | 0.12 | 0.81 |
|  | EH | 66.64 | 12.04 | 27.73 | 0.63 | 0.11 | 0.74 |
|  | RNPE | 0.55 | 0.14 | 0.26 | 0.58 | 0.14 | 0.73 |
|  | KNPR | 4.06 | 1.84 | 6.01 | 0.34 | 0.15 | 0.50 |
|  | KT | 8.18 | 3.60 | 6.06 | 0.46 | 0.20 | 0.66 |
|  | KW | 11.90 | 3.60 | 6.58 | 0.54 | 0.16 | 0.70 |
|  | KL | 10.58 | 7.77 | 8.79 | 0.39 | 0.29 | 0.68 |
|  | VW | 285.21 | 224.63 | 648.60 | 0.25 | 0.19 | 0.44 |
|  | HGW | 4.86 | 1.77 | 3.07 | 0.50 | 0.18 | 0.68 |
|  | GY | 49.94 | 31.62 | 66.97 | 0.34 | 0.21 | 0.55 |
| TC | PH | 53.06 | 12.89 | 22.14 | 0.60 | 0.15 | 0.75 |
|  | EH | 32.33 | 5.43 | 10.52 | 0.67 | 0.11 | 0.78 |
|  | RNPE | 0.40 | 0.06 | 0.12 | 0.70 | 0.10 | 0.80 |
|  | KNPR | 1.27 | 0.52 | 1.17 | 0.43 | 0.17 | 0.60 |
|  | KT | 2.25 | 0.50 | 0.89 | 0.62 | 0.14 | 0.75 |
|  | KW | 6.09 | 1.35 | 2.57 | 0.61 | 0.13 | 0.74 |
|  | KL | 7.57 | 1.74 | 5.91 | 0.50 | 0.11 | 0.61 |
|  | VW | 156.13 | 103.25 | 248.62 | 0.31 | 0.20 | 0.51 |
|  | HGW | 1.84 | 0.68 | 1.10 | 0.51 | 0.19 | 0.70 |
|  | GY | 36.52 | 16.67 | 49.86 | 0.35 | 0.16 | 0.52 |
| TM | PH | 45.56 | 15.87 | 22.59 | 0.54 | 0.19 | 0.73 |
|  | EH | 38.37 | 5.45 | 9.26 | 0.72 | 0.10 | 0.83 |
|  | RNPE | 0.21 | 0.03 | 0.07 | 0.68 | 0.10 | 0.78 |
|  | KNPR | 2.87 | 0.96 | 1.61 | 0.53 | 0.18 | 0.70 |
|  | KT | 3.37 | 0.66 | 1.30 | 0.63 | 0.12 | 0.76 |
|  | KW | 5.34 | 1.17 | 2.25 | 0.61 | 0.13 | 0.74 |
|  | KL | 7.49 | 1.92 | 3.83 | 0.57 | 0.15 | 0.71 |
|  | VW | 170.47 | 76.95 | 195.46 | 0.38 | 0.17 | 0.56 |
|  | HGW | 1.92 | 0.58 | 1.08 | 0.54 | 0.16 | 0.70 |
|  | GY | 34.73 | 15.56 | 31.83 | 0.42 | 0.19 | 0.61 |

$h_{a}^{2}$, the proportion of phenotypic variance contributed by additive variance (narrow-sense heritability), calculated by $h_{a}^{2}=\frac{\sigma_{a}^{2}}{(\sigma_{a}^{2}+\sigma_{aa}^{2}+\sigma_{\varepsilon}^{2})}$;

$h_{aa}^{2}$, the proportion of phenotypic variance contributed by additive-by-additive variance, calculated by $h_{aa}^{2}=\frac{\sigma_{aa}^{2}}{(\sigma_{a}^{2}+\sigma_{aa}^{2}+\sigma_{\varepsilon}^{2})}$;

$h^{2}$, broad-sense heritability, calculatd by $h^{2}=\frac{\sigma_{a}^{2}+\sigma_{aa}^{2}}{(\sigma_{a}^{2}+\sigma_{aa}^{2}+\sigma_{\varepsilon}^{2})}$.

PH, plant height; EH, ear height; RNPE, row number per ear; KNPR, kernel number per row; KT, kernel thickness; KW, kernel width; KL, kernel length; VW, volume weight; HGW, hundred grain weight; GY, grain yield per plant.
