# Supplementary material for: Genetic Dissection of Hybrid Performance and Heterosis for Yield-Related Traits in Maize
Source: Front Plant Sci. 2021 Nov 30;12:774478. doi: 10.3389/fpls.2021.774478 (PMC8670227; doi:10.3389/fpls.2021.774478)
Supplement: Supplementary Table 6 — Variance components and proportion of the phenotypic variance contributed by each variance component in the pooled Chang7-2 × RIL (TC)-Mo17 × RIL (TM) population. [file Table_6.docx]

**Supplementary Table 6 |** Variance components and proportion of the phenotypic variance contributed by each variance component in the pooled Chang7-2 × RIL (TC)- Mo17 × RIL (TM) population

|  | PH | EH | RNPE | KNPR | KT | KW | KL | VW | HGW | GY |
| --- | --- | --- | --- | --- | --- | --- | --- | --- | --- | --- |
| $\sigma_{a}^{2}$ | 18.06 | 28.23 | 0.14 | 0.65 | 1.84 | 1.34 | 3.77 | 98.47 | 0.63 | 13.9 |
| $\sigma_{d}^{2}$ | 42.94 | 33.73 | 0.37 | 3.15 | 2.15 | 9.65 | 12.9 | 138.38 | 4.5 | 40.74 |
| $\sigma_{aa}^{2}$ | 66.92 | 36.19 | 0.18 | 1.64 | 2.7 | 2.69 | 3.24 | 133.13 | 0.97 | 23.45 |
| $\sigma_{ad}^{2}$ | 64.2 | 19.8 | 0.15 | 1.5 | 1.9 | 2.6 | 3.83 | 157.59 | 0.87 | 20.19 |
| $\sigma_{dd}^{2}$ | 37.45 | 13.52 | 0.12 | 1.5 | 1.68 | 3.26 | 4.82 | 127.53 | 0.99 | 23.08 |
| $\sigma_{\varepsilon}^{2}$ | 13.53 | 7.39 | 0.11 | 1.61 | 1.1 | 2.6 | 4.91 | 239.9 | 1.16 | 49.45 |
| $h_{a}^{2}$ | 0.07 | 0.20 | 0.13 | 0.06 | 0.16 | 0.06 | 0.11 | 0.11 | 0.07 | 0.08 |
| $h_{d}^{2}$ | 0.18 | 0.24 | 0.34 | 0.31 | 0.19 | 0.44 | 0.39 | 0.15 | 0.49 | 0.24 |
| $h_{aa}^{2}$ | 0.28 | 0.26 | 0.16 | 0.16 | 0.24 | 0.12 | 0.10 | 0.15 | 0.11 | 0.14 |
| $h_{ad}^{2}$ | 0.26 | 0.14 | 0.14 | 0.15 | 0.17 | 0.12 | 0.11 | 0.18 | 0.10 | 0.12 |
| $h_{dd}^{2}$ | 0.15 | 0.10 | 0.11 | 0.15 | 0.15 | 0.15 | 0.14 | 0.14 | 0.11 | 0.14 |

All genetic variance $\sigma^{2}$ is calculated by $\sigma^{2}$ = $\sigma_{a}^{2}+\sigma_{d}^{2}+\sigma_{aa}^{2}+h_{ad}^{2}+h_{dd}^{2}+\sigma_{\varepsilon}^{2}$;

$h_{a}^{2}$, the genetic proportion of additive effect, calculated by $h_{a}^{2}=\sigma_{a}^{2}/\sigma^{2}$;

$h_{d}^{2}$, the genetic proportion of dominance effect, calculated by $h_{d}^{2}=\sigma_{d}^{2}/\sigma^{2}$;

$h_{aa}^{2}$, the genetic proportion of additive-by-additive effect, calculated by $h_{aa}^{2}=\sigma_{aa}^{2}/\sigma^{2}$;

$h_{ad}^{2}$, the genetic proportion of additive-by-dominance effect, calculated by $h_{ad}^{2}=\sigma_{ad}^{2}/\sigma^{2}$;

$h_{dd}^{2}$, the genetic proportion of dominance-by-dominance effect, calculated by $h_{dd}^{2}=\sigma_{dd}^{2}/\sigma^{2}$.

PH, plant height; EH, ear height; RNPE, row number per ear; KNPR, kernel number per row; KT, kernel thickness; KW, kernel width; KL, kernel length; VW, volume weight; HGW, hundred grain weight; GY, grain yield per plant.
