# Supplementary material for: Genetic Dissection of Hybrid Performance and Heterosis for Yield-Related Traits in Maize
Source: Front Plant Sci. 2021 Nov 30;12:774478. doi: 10.3389/fpls.2021.774478 (PMC8670227; doi:10.3389/fpls.2021.774478)
Supplement: Supplementary Table 7 — Variance components and proportion of the phenotypic variance contributed by each variance component for midparent heterosis. [file Table_7.DOCX]

**Supplementary Table 7 |** Variance components and proportion of the phenotypic variance contributed by each variance component for midparent heterosis

|  | PH | EH | RNPE | KNPR | KT | KW | KL | VW | HGW | GY |
| --- | --- | --- | --- | --- | --- | --- | --- | --- | --- | --- |
| $\sigma_{d}^{2}$ | 71.70 | 29.00 | 0.15 | 4.21 | 0.72 | 2.89 | 7.40 | 98.16 | 0.56 | 75.61 |
| $\sigma_{aa}^{2}$ | 43.79 | 18.42 | 0.12 | 2.15 | 4.29 | 1.64 | 2.01 | 95.79 | 0.91 | 10.28 |
| $\sigma_{ad}^{2}$ | 48.07 | 9.47 | 0.13 | 1.13 | 5.36 | 3.26 | 3.18 | 145.93 | 1.13 | 19.33 |
| $\sigma_{dd}^{2}$ | 26.65 | 6.77 | 0.06 | 0.82 | 0.97 | 2.24 | 2.54 | 112.20 | 0.75 | 13.60 |
| $\sigma_{\varepsilon}^{2}$ | 29.36 | 9.07 | 0.17 | 1.56 | 1.26 | 7.72 | 8.50 | 460.65 | 1.31 | 48.73 |
| $h_{d}^{2}$ | 0.33 | 0.40 | 0.24 | 0.43 | 0.06 | 0.16 | 0.31 | 0.11 | 0.12 | 0.45 |
| $h_{aa}^{2}$ | 0.20 | 0.25 | 0.18 | 0.22 | 0.34 | 0.09 | 0.08 | 0.10 | 0.20 | 0.06 |
| $h_{ad}^{2}$ | 0.22 | 0.13 | 0.21 | 0.11 | 0.43 | 0.18 | 0.13 | 0.16 | 0.24 | 0.12 |
| $h_{dd}^{2}$ | 0.12 | 0.09 | 0.10 | 0.08 | 0.08 | 0.13 | 0.11 | 0.12 | 0.16 | 0.08 |

All genetic variance $\sigma^{2}$ of midparent heterosis is calculated by $\sigma^{2}$ = $\sigma_{d}^{2}+\sigma_{aa}^{2}+\sigma_{ad}^{2}+\sigma_{dd}^{2}+\sigma_{\varepsilon}^{2}$;

$h_{d}^{2}$, the genetic proportion of dominance effect, calculated by $h_{d}^{2}=\sigma_{d}^{2}/\sigma^{2}$;

$h_{aa}^{2}$, the genetic proportion of additive-by-additive effect, calculated by $h_{aa}^{2}=\sigma_{aa}^{2}/\sigma^{2}$;

$h_{ad}^{2}$, the genetic proportion of additive-by-dominance effect, calculated by $h_{ad}^{2}=\sigma_{ad}^{2}/\sigma^{2}$;

$h_{dd}^{2}$, the genetic proportion of dominance-by-dominance effect, calculated by $h_{dd}^{2}=\sigma_{dd}^{2}/\sigma^{2}$.

PH, plant height; EH, ear height; RNPE, row number per ear; KNPR, kernel number per row; KT, kernel thickness; KW, kernel width; KL, kernel length; VW, volume weight; HGW, hundred grain weight; GY, grain yield per plant.
