# Supplementary material for: Genetic Dissection of Hybrid Performance and Heterosis for Yield-Related Traits in Maize
Source: Front Plant Sci. 2021 Nov 30;12:774478. doi: 10.3389/fpls.2021.774478 (PMC8670227; doi:10.3389/fpls.2021.774478)
Supplement: Supplementary Table 9 — Correlations between the number of harbored favorable quantitative trait loci (QTL) and hybrid performance for 10 traits in Chang7-2 × RIL (TC) and Mo17 × RIL (TM) population, respectively. [file Table_9.docx]

**Supplementary Table 9 |** Correlations between the number of harbored favorable quantitative trait loci (QTL) and hybrid performance for 10 traits in Chang7-2 × RIL (TC) and Mo17 × RIL (TM) population, respectively

| Population | Traits | *r*_1_ | *r*_2_ | *r*_3_ |
| --- | --- | --- | --- | --- |
| TC | PH | 0.29^**^ | 0.44^**^ | 0.51^**^ |
|  | EH | 0.49^**^ | 0.13^*^ | 0.48^**^ |
|  | RNPE | 0.20^**^ | 0.45^**^ | 0.48^**^ |
|  | KNPR | 0.26^**^ | / | 0.26^**^ |
|  | KT | 0.25^**^ | 0.43^**^ | 0.47^**^ |
|  | KW | 0.35^**^ | 0.23^**^ | 0.40^**^ |
|  | KL | 0.30^**^ | 0.30^**^ | 0.41^**^ |
|  | VW | 0.37^**^ | 0.26^**^ | 0.45^**^ |
|  | HGW | 0.19^**^ | 0.49^**^ | 0.52^**^ |
|  | GY | 0.16^**^ | 0.43^**^ | 0.41^**^ |
| TM | PH | 0.33^**^ | 0.24^**^ | 0.39^**^ |
|  | EH | 0.32^**^ | 0.35^**^ | 0.48^**^ |
|  | RNPE | 0.35^**^ | 0.21^**^ | 0.36^**^ |
|  | KNPR | 0.20^**^ | 0.24^**^ | 0.30^**^ |
|  | KT | 0.30^**^ | 0.46^**^ | 0.51^**^ |
|  | KW | 0.36^**^ | / | 0.36^**^ |
|  | KL | 0.38^**^ | 0.13^*^ | 0.37^**^ |
|  | VW | / | 0.33^**^ | 0.33^**^ |
|  | HGW | 0.46^**^ | 0.40^**^ | 0.54^**^ |
|  | GY | 0.42^**^ | 0.25^**^ | 0.47^**^ |

*r_1_*, the correlation between the number of favorable homozygous QTL and hybrid performance in TC and TM;

*r_2_*, the correlation between the number of favorable heterozygous QTL in TM and hybrid performance;

*r_3_*, the correlation between the number of all favorable QTL in TM and hybrid performance.

*, significant at the 0.05 level; **, significant at 0.01 level; /, the correlations could not be calculated because the corresponding QTL could not be found. PH, plant height; EH, ear height; RNPE, row number per ear; KNPR, kernel number per row; KT, kernel thickness; KW, kernel width; KL, kernel length; VW, volume weight; HGW, hundred grain weight; GY, grain yield per plant.
